# True gender ratios and stereotype rating norms

**DOI:** 10.3389/fpsyg.2015.01023

**Published:** 2015-07-22

**Authors:** Alan Garnham, Sam Doehren, Pascal Gygax

**Affiliations:** ^1^School of Psychology, University of SussexBrighton, UK; ^2^Department of Psychology, University of FribourgFribourg, Switzerland

**Keywords:** stereotypes, role names, ratings, true ratios, archival sources

## Abstract

We present a study comparing, in English, perceived distributions of men and women in 422 named occupations with actual real world distributions. The first set of data was obtained from previous a large-scale norming study, whereas the second set was mostly drawn from UK governmental sources. In total, real world ratios for 290 occupations were obtained for our *perceive* vs. *real world* comparison, of which 205 were deemed to be unproblematic. The means for the two sources were similar and the correlation between them was high, suggesting that people are generally accurate at judging real gender ratios, though there were some notable exceptions. Beside this correlation, some interesting patterns emerged from the two sources, suggesting some response strategies when people complete norming studies. We discuss these patterns in terms of the way real world data might complement norming studies in determining gender stereotypicality.

## Introduction

Gender stereotyping forms a cornerstone of psychology with many sub-domains researching the topic in detail, in particular, and for very different reasons, social psychology and psycholinguistics. The study of gender stereotyping in social psychology focuses on the processes that lead to stereotyping – applying a set of beliefs about the characteristics of a social category to members of that category (Greenwald and Banaji, 1995) – and the wider result of this stereotyping; see, for example, Peterson and Zurbriggen (2010) and Latrofa et al. (2012). Gender stereotyping in psycholinguistics has typically been studied as an example of inference in the comprehension of discourse and text. When an individual is described as an engineer, researchers have been interested in possible inferences about the gender of this engineer. Much of this research uses anaphor resolution as in index of stereotyping (e.g., Carreiras et al., 1996) or judgments about words that explicitly or implicitly refer to a person of a given gender (e.g., Oakhill et al., 2005; Gygax and Gabriel, 2008).

In this psycholinguistic literature, stereotyped words are often compared and contrasted with words with definitional gender, such as *king* and *queen* (e.g., Banaji and Hardin, 1996; Osterhout et al., 1997; Oakhill et al., 2005). As in the case of *king* and *queen*, these words often form morphologically unrelated pairs. Morphologically related pairs, such as actor and actress, have undergone considerable changes in usage over the past 50 years. The definitions of role names that are gendered by stereotype do not contain gender information as part of their core meaning, which defines the role itself (what a footballer does, for example, or a secretary). It therefore follows that if the effect of the gender stereotypicality of a noun, or a role name (e.g., *taxi driver*) more generally, is to be studied, the extent of the stereotyping of the noun first needs to be measured.

Because psycholinguistic studies of stereotyping look at whether, or how likely, an inference about a person’s gender will be made on the basis of stereotype information, it has been considered appropriate to assess the proportions of men and women thought, by people similar to those tested in the core experiment, to fill various roles in the real world. In collecting stereotype norms, therefore, the method has been to collate a set of role names (which may be either single nouns, such as *nurse*, or phrases such as *primary school teacher*), present them to judges, and use a variant of the instruction: estimate to what extent the groups are made up of women or men. Data is typically collected on a Likert-type scale (e.g., Kennison and Trofe, 2003; Gabriel et al., 2008; Irmen and Kurovskaja, 2010; Misersky et al., 2014). These studies have often been carried out as pre-tests for a particular further study, rather than as studies in their own right. Misersky et al. (2014) pointed out that, therefore, the methods have varied enough to prevent direct comparison between studies. The study carried out by Misersky et al. (2014) used a common data collection tool, designed for the study but extensible to other languages, to collect stereotype norms in seven languages and for a large set of role names. Four hundred and twenty-two role names were chosen to be tested for English, and as many of those in the other six languages that had translations from English. Selection was based on previous norming studies (e.g., Kennison and Trofe, 2003; Gabriel et al., 2008), as well as on brainstorming sessions and trawls of dictionaries. All of the chosen terms were intended to be stereotypically applied to males or females, but not definitionally. This distinction is not always completely clear-cut, partly because of changing matters of usage. A particularly tricky case is *waiter*, which was once part of a gender marked pair *waiter/waitress*, and was the subject in the United States of a largely failed attempt to replace it with the supposedly gender neutral term *server*. The Cambridge free English dictionary (Cambridge Dictionaries Online, 2015), for example, defines a waiter as “a man whose job is to bring the food to customers at their tables in a restaurant,” though other sources reflect more progressive thinking (Cambridge Dictionaries Online, 2015, under *Sexist language*). In the Misersky et al. (2014) study, respondents were free to indicate that they thought 100% of waiters were male, though the actual figure was 45%, and the true data from ONS sources suggested 75%. As in previous studies carried out by our group (e.g., Gabriel et al., 2008), an 11-point Likert type scale was used, ranging from 0% women/100% men to 100% women/0% men, in 10% steps, and participants were asked to estimate to what extent the roles presented to them were carried out by women or men. Participants were specifically asked to think of the *real* proportion of men and women in the roles (and not to base their responses on how they thought things should be). Data were collected online, and in the English sample there were 281 respondents, far more than in previous studies and hence providing reasonably accurate estimates of beliefs about the proportions of men and women filling the roles studied (see original paper for data).

Though stereotyping is often seen as a negative and prejudicial activity, it is widely accepted as a required process for simplifying a complex world via the use of schemas (Augoustinos and Walker, 1998; López-Sáez et al., 2008; Wilbourn and Kee, 2010). Within the social psychology literature, attempts have been made to determine whether stereotyping is based on outdated true gender bias (Wilbourn and Kee, 2010), or (possibly incorrect) assumptions about current female/male ratios (Lopez-Zafra and Garcia-Retamero, 2012; Mills et al., 2012). However, exact gender ratios are not usually reported, so the conclusions can be difficult to evaluate. In the psycholinguistic domain, it is sensible to assume that comprehension is driven by beliefs about male/female ratios, rather than unknown (to the comprehender) true ratios. Nevertheless, the question can be asked about the relation between assumed and true ratios. The answer to that question bears both on the interpretation of psycholinguistic findings, and also, more importantly in the present context, potential prejudice based on completely incorrect assumptions. The current study, therefore, aims to provide true gender ratios for as many of the English role names that appear in the Misersky et al. (2014) study as possible, and to compare them with the reported ratios in the Misersky et al. (2014) data set. Because of the lack of previous research on true gender ratios it is an open question how closely related the norm data and true gender ratios will be.

The main source of information about true gender ratios was, where possible, archival data collected by the UK Office of National Statistics (ONS, http://www.ons.gov.uk/). Where necessary other archival resources were used. The primary objective the current research is, therefore, to collect true gender ratios for the role names presented in Misersky et al. (2014), and to compare them with the normative data from that study.

## Materials and Methods

We used archival data to collect true gender ratios for as many as possible of the 422 English role descriptions from Misersky et al. (2014), reproduced in data sheet 1 in the supplementary material.

The data were primarily collected from governmental, in particular the UK Office for National Statistics (ONS), and academic sources. In a minority of cases other sources were considered appropriate, and were used. Where no source was available, or considered to be reliable, no estimate of the true ratio was obtained.

The archive search had a number of stages, and proceeded on an item-by-item basis, rather than a source-by-source basis. An attempt was made to locate each item in each source in order. If a source failed to provide relevant data, the next source was consulted. If relevant data were found at any stage, the process ended and the next source was not searched. If the mapping between a role name in the Misersky norms and information in a source was unclear, supplementary information on governmental and academic sites was used to clarity the definition of the role name in the archival data (no definitions were provided in the normative study). The ONS Standard Occupational Classification (ONS, 2010) was the most important document in this context. On occasion more than one definition was available. In such cases, all definitions were incorporated, if possible.

The process and sources were as follows:

(1) 2011 Census, Population Estimates by single year of age and sex for Local Authorities in the UK (ONS, 2013a)
  (i) This source is a list of demographic information about age and gender of the population of the UK.

(2) Reference table EMP16 ‘Employment by occupation’ (ONS, 2013c), in conjunction with the Standard Occupational Classification 2010 Volume 2 The coding index (ONS, 2010)
  (i) Reference table EMP16 is a list of general job roles with the numbers of people from each gender that perform that role as an occupation, both full and part time.
  (ii) The Standard Occupational Classification coding index is a detailed list of job roles and provides the four-level classification ONS uses in EMP16.
    (I) The Standard Occupational Classification (SOC) coding index was searched for the role name; all occurrences of the role name were used. This search provided a list of ‘SOC’ codes that were cross-referenced with EMP16 to provide the gender ratios.
    (II) If two, or more, job roles returned the same SOC code for one role name, each SOC code was only used once to estimate the gender ratio for each role name.

(3) Other UK governmental sources
  (i) A Google search was performed with the role name combined with the search terms ‘gender statistics’ and ‘gender ratio’ to find appropriate websites sources.
  (ii) Only sites with UK governmental top-level domains were accepted at this stage; for example, .gov.uk or .mod.uk.
    (I) Sports based role names were the exception to this rule; statistics obtained directly from governing bodies were accepted if UK specific statistics were provided; as was the case, for example, for the Football Association.

(4) Academic sources
  (i) Scopus and Google Scholar were searched for the role names with, and without, the addition of the phrases ‘gender statistics’ and ‘gender ratio.’

(5) Other sources
  (i) As with ‘Other UK governmental sources,’ a Google search was performed with the role name combined with search terms ‘gender statistics’ and ‘gender ratio’ to find appropriate website sources.
  (ii) Each source was judged on its own merits; for example, national UK news sources and national bodies were accepted, but blogs were not.

Each ratio was assessed for quality. The first criterion for quality was recency. Ratios dated prior to 2008 (5 years prior to the work being carried out) were marked as questionable. Only one ratio was considered questionable on these grounds. Initially recency was to be the only criterion for the quality of the ratios, as the quality of the sources was supposed to be guaranteed by the collection process. However, during the process of data collection a second set of issues became apparent in the ratios produced from the ONS employment data (stage 2, above). The process of collating the list of SOC codes from the Standard Occupational Classification (ONS, 2010) involved identifying all occurrences of the relevant role name in the list, and it produced two types of problem. First, a specific term in Misersky et al.’s (2014) list was only located in one broader category. For example, the role name ‘Zoologists’ was deemed to be part of the job role ‘Biological scientists and biochemists,’ which covers more than just ‘Zoologists.’ Second, a single term in Misersky et al.’s (2014) list was associated with a large number of job roles. For example the role name ‘Manager’ was part of 1336 job descriptions, which were associated with 121 different SOC codes. In such cases, it is not clear that Misersky et al.’s (2014) participants would have all these possibilities in mind when making their judgments. Therefore, if the job role was deemed too broad, or if it was associated with more than ten SOC codes, the resulting ratio was classified as questionable.

We found archival data on true gender ratios for 290 (out of 422) of the role names in Misersky et al.’s (2014) English list. As can be seen in **Table 1**, the vast majority of the true gender ratios were found in stage 2 of the archival search process, though many of these have been classified as questionable. In total, 86 ratios of the 290 ratios have been so classified. The stage where each questionable ratio was collected is shown in **Table 1**, and the role names with questionable ratios are flagged in data sheet 1 in the supplementary material.

**Table 1:** List of data collection stages.

Stage	Role names	Questionable
1	17 (4.03%)	0
2	230 (54.5%)	84
3	30 (7.11%)	1
4	2 (0.47%)	1
5	11 (2.61%)	0
No data	132 (31.28%)	NA

The 132 role names for which no data have been found include about 20 cases where data are unlikely to be obtainable. Some roles, such as ‘Executioners’ no longer exist in British society, others are difficult to define or collect data for (e.g., ‘Clients’), and others may be protected by considerations of security (e.g., ‘Spies”). For the rest, data are in principle obtainable, though possibly from sources that would be unreliable.

## Results

The mean true gender ratio of the 290 role names was 0.44 (SD = 0.17), where 1.00 would represent 100% females and, 0.00, 100% males. This mean is similar to the mean found in Misersky et al. (2014) for the same role names (*M* = 0.43, SD = 0.30). The range of the ratios was 0.00 to 1.00, this compares to the Misersky et al. (2014) range of 0.15 to 0.84. Skew and kurtosis were modest, 0.49 and -0.68, respectively.

A two-tailed Pearson’s correlation was calculated to investigate how the findings of Misersky et al. (2014) related to the true gender ratios collected in this study. It was found that there was a strong significant positive relationship between the two data sets (*r* = 0.755, *N* = 290, *p* < 0.001).

As many of the ratios had been highlighted as questionable during the collection process, it was decided to separate these ratios from the non-questionable data and perform a Pearson’s correlation on each set separately. Removing the questionable ratios improved the correlation (*r* = 0.849, *N* = 205, *p* < 0.001). The questionable ratios also correlated significantly with the relevant judged ratios, though much less strongly (*r* = 0.273, *N* = 85, *p* = 0.011).

**FIGURE 1:**
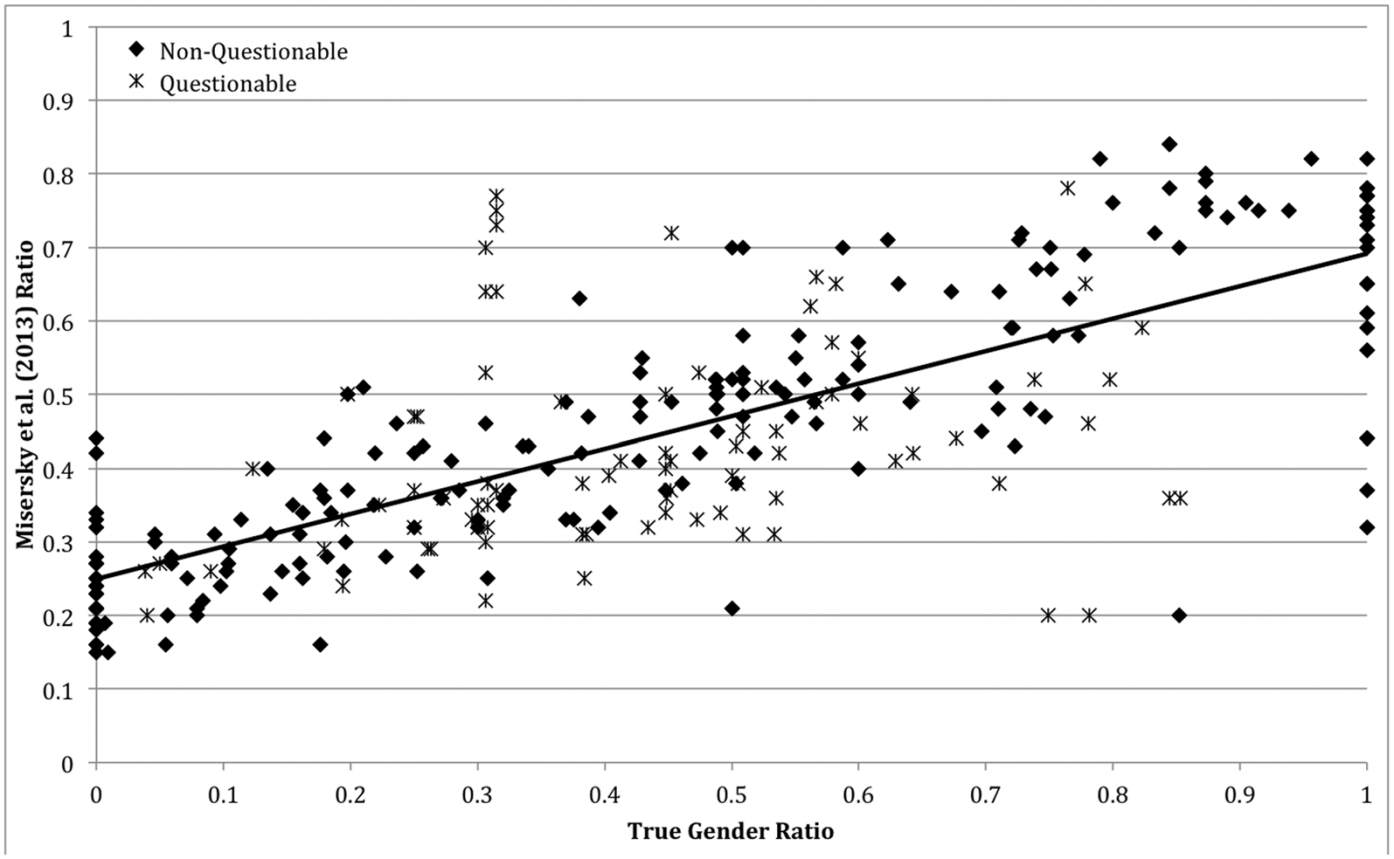
**Scatter plot of real gender ratios from current study against normative judgments from Misersky et al. (2014).** The solid line is the line of best fit for all data.

**Figure 1** highlights the difference in the range of the ratios found in the two studies, as well as separately indicating the questionable and non-questionable ratios. Numerical values for all the ratios can be found in data sheet 1 in the supplementary material.

## Discussion

The primary aim of this study was to produce true gender ratios for as many as possible of the 422 English role names for which Misersky et al. (2014) reported judgments of gender ratio. These true gender ratios were to be compared with the normative judgments reported by Misersky et al. (2014). To date there has been relatively little study of true gender ratios, and none on the scale of the current survey.

The overall mean in this study (0.44) suggests a tendency for the role names selected to be predominantly male. This fact may be explained by the fact that majority of the true gender ratios are for occupational role names and ONS (2014) states that the majority of workers are male, with a true gender ratio of 0.47. Figures from the past would show a greater proportion of males in the UK workforce. Full details of true gender ratios for individual role names are available in data sheet 1 in the supplementary material.

The second aim of this study was to compare the true gender ratios with the normative judgment data on stereotypicality from Misersky et al. (2014). The two studies found similar means for the gender ratio across the 290 role names for which both types of data were available (current study, *M* = 0.44, SD = 0.17; previous study, *M* = 0.43, SD = 0.30). Misersky et al. (2014) attribute this male bias to stronger male stereotypes, as did a previous study that was similar in nature (Gabriel et al., 2008). This study, looking at true gender ratios, found a similar mean to Misersky et al. (2014), Rather than suggesting stronger male stereotypes, in any sense suggesting a mismatch with reality, it appears that the role names investigated refer to roles that, on average, more males than females fill. Looking at the role names, it is clear that the majority of them are occupations, or could be viewed as occupations, and, as previously mentioned, the work force in the UK is predominantly male. It would, therefore, be expected that there would be a slight male bias (ONS, 2014).

As well as finding similar means, the two studies produced data for the 290 roles names that are significantly correlated (*r* = 0.755, *p* < 0.001). This correlation improved when the ratios classified as questionable were removed from the analysis (*r* = 0.849, *N* = 205, *p* < 0.001). The two findings together, means and correlation, suggests that people are generally able to provide an accurate estimate of the true gender ratio for a role name.

Though people were generally correct in their estimates of gender ratios, there were exceptions. For a small number of role names, the discrepancy between the estimate and the true gender ratio was over 0.50. These role names were: Tailor, Barber, Probation Officer, Hunter, Archivist, Curator, and Butler. All discrepancies over 0.50 involved overestimation of the proportion of males who performed the role. This fact provides some limited support for the conclusion drawn by Gabriel et al. (2008) and Misersky et al. (2014) that male stereotypes are stronger than female stereotypes. However, except for Barber, the true gender ratios for the seven roles names in this category are considered questionable, four for having absolute values of 1.00 (see below) and the remaining two because their SOC code refers to an overly broad category.

As mentioned in the methods section, some of the ratios are classified as questionable (for our purposes) because the source provides information about a similar, but not the same, role name as the one we believe people were making judgments about. For example, in the ONS data, ‘Author’ was included in the broader categories “Authors, writers and translators” and “Programmers and software development professionals,” with no possibility of disaggregating the data. It is unlikely that Misersky et al.’s (2014) participants had this definition of ‘Author’ in mind when making their judgments.

In addition, as can be seen in **Figure 1**, a number of role names (53) have ratios of 0.00 (all men) or 1.00 (all women). Two of these ratios, ‘Admirals’ [all men, DASA (Navy) (2013)] and ‘Synchronized swimmers’ (all women, Fédération Internationale de Natation, 2013), came from stage 3 of the collection process. Both of these ratios came from reliable sources and are accepted as correct. The remaining 51 of these ratios came from stage 2 of the collection process and reflect the fact that the number of workers of one gender is considered “too small for reliable estimate,” and so cannot be distinguished from zero (ONS, 2013c). In EMP16 (ONS, 2013c) no information is given about what counts as too small. However, it can be inferred that the cut off for this classification occurs between 0 and 4713 people occupying the role, this number being one less than the lowest statistic that is provided for any job role. The effect on the resultant ratio varies considerably between role names. For example, 470,749 males are said to be ‘Electricians,’ whereas the number females is “too small for reliable estimate.” In this case, even if there were 4713 females electricians, the ratio would only change from 0.00 to 0.01. ‘Shoemakers,’ on the other hand, also has a 0.00 ratio, but with only 6305 males; in this case the potential change from including 4713 women is from 0.00 to 0.43.

Another issue arises from the use, by Misersky et al. (2014), of an 11-point Likert scale with 10% increments for the estimation of ratios. Participants might be reluctant to use extreme values (0% men, 0% women) when they know that some women or men do occupy certain roles. They might have been less reluctant to provide values closer to 0 or 100% on a less coarse scale, though the issue of whether sliders are preferable to radio button/Likert-type scales is a complex one (Cook et al., 2001). Another reason why participants might be reluctant to use extreme values could be that they try to produce socially desirable responses, and hence avoid extreme values, to look open minded. Although the instructions did ask participants to dissociate themselves from their view of gender equality, we cannot be sure to what extent they followed this instruction.

The true gender ratios collected as part of this study should aid future research on stereotyping. Not only do they provide a detailed catalog of true gender ratios. They also allow a distinction to be drawn between stereotyped role names that are correctly judged to be typical of one gender and those that are not. The question of why some occupations are typical of one gender still remains, but the question of why some estimates are better than others is an interesting one for future research and researchers may well want to consider their data set in terms of how big the discrepancy is between stereotype beliefs and true typicality.

One issue that neither the current, nor previous, research has addressed is the familiarity of the role names. It is reasonable to assume that the more familiar a person is with a role name, the more likely it is that they will have specific knowledge related to that role, including knowledge of true gender ratios. There are at least two different ways to incorporate questions about familiarity into research of this kind. First, the data collection tool developed for the Misersky et al. (2014) study could be augmented to collect familiarity information. Second, Blair et al. (2002) found that estimates of word frequency using Internet search methods correlate reasonably well with familiarity ratings. This second method would not be as satisfactory, as it would not provide direct estimates of familiarity. However, it could produce results more quickly, and might be preferred for that reason.

## Conflict of Interest Statement

The authors declare that the research was conducted in the absence of any commercial or financial relationships that could be construed as a potential conflict of interest.
