# Supplementary material for: True gender ratios and stereotype rating norms
Source: Front Psychol. 2015 Jul 22;6:1023. doi: 10.3389/fpsyg.2015.01023 (PMC4510832; doi:10.3389/fpsyg.2015.01023)
Supplement: Supplementary file 1 [file Data_Sheet_1.DOCX]

| **Data Sheet 1** | | | | | | | |  | | | |  | | |  | | | |  |  | |  | |  | |  |  | | |  |
| --- | --- | --- | --- | --- | --- | --- | --- | --- | --- | --- | --- | --- | --- | --- | --- | --- | --- | --- | --- | --- | --- | --- | --- | --- | --- | --- | --- | --- | --- | --- |
| List of Role names with True Gender Ratio, Misersky et al. (2014) Ratio, Source, Whether Questionable, and Approximate Raw Numbers | | | | | | | | | | | | | | | | | | | | | | | | | | | | |  |  |
| Role Noun | True Ratio | | Misersky et al. (2013) Ratio | | Ratio difference | | Source | | Questionable? | | Total number from the archive search | | | | Number of *men* from the archive search | | Number of *women* from the archive search |  |  |  |  |  |  |  |  |  |  |  |  |  |
| Accountants | 0,32 | | 0,37 | | 0,05 | | 1 | |  | | 312 104 | | | | 210 824 | | 101 280 |  |  |  |  |  |  |  |  |  |  |  |  |  |
| Acrobats | - | | 0,57 | |  | |  | |  | | 0 | | | | 0 | | 0 |  |  |  |  |  |  |  |  |  |  |  |  |  |
| Acupuncturists | 0,82 | | 0,59 | | -0,23 | | 2 | | Yes | | 87 540 | | | | 15 483 | | 72 057 |  |  |  |  |  |  |  |  |  |  |  |  |  |
| Administrative workers | 0,72 | | 0,59 | | -0,13 | | 2 | |  | | 2 506 554 | | | | 702 400 | | 1 804 154 |  |  |  |  |  |  |  |  |  |  |  |  |  |
| Admirals | 0 | | 0,25 | | 0,25 | | 3 | |  | | 40 | | | | 40 | | 0 |  |  |  |  |  |  |  |  |  |  |  |  |  |
| Aerobics instructors | 0,59 | | 0,7 | | 0,11 | | 2 | |  | | 54 518 | | | | 22 479 | | 32 039 |  |  |  |  |  |  |  |  |  |  |  |  |  |
| Air traffic controllers | 0 | | 0,32 | | 0,32 | | 2 | |  | | 6 675 | | | | 6 675 | | 0 |  |  |  |  |  |  |  |  |  |  |  |  |  |
| Alcoholics | 0,28 | | 0,41 | | 0,13 | | 4 | |  | | 57 600 | | | | 41 500 | | 16 100 |  |  |  |  |  |  |  |  |  |  |  |  |  |
| Anglers | - | | 0,28 | |  | |  | |  | | 0 | | | | 0 | | 0 |  |  |  |  |  |  |  |  |  |  |  |  |  |
| Animators | 0,5 | | 0,39 | | -0,11 | | 2 | | Yes | | * | | | | * | | * |  |  |  |  |  |  |  |  |  |  |  |  |  |
| Antique dealers | 0,38 | | 0,38 | | 0 | | 2 | | Yes | | 156 823 | | | | 96 867 | | 59 956 |  |  |  |  |  |  |  |  |  |  |  |  |  |
| Apprentices | - | | 0,4 | |  | |  | |  | | 0 | | | | 0 | | 0 |  |  |  |  |  |  |  |  |  |  |  |  |  |
| Architects | 0,27 | | 0,36 | | 0,09 | | 2 | |  | | 58 322 | | | | 42 494 | | 15 828 |  |  |  |  |  |  |  |  |  |  |  |  |  |
| Archivists | 1 | | 0,44 | | -0,56 | | 2 | |  | | 8 749 | | | | 0 | | 8 749 |  |  |  |  |  |  |  |  |  |  |  |  |  |
| Artists | 0,54 | | 0,51 | | -0,03 | | 2 | |  | | 51 741 | | | | 24 048 | | 27 693 |  |  |  |  |  |  |  |  |  |  |  |  |  |
| Assistant chefs | 0,68 | | 0,44 | | -0,24 | | 2 | | Yes | | 436 111 | | | | 140 858 | | 295 253 |  |  |  |  |  |  |  |  |  |  |  |  |  |
| Assistants | 0,58 | | 0,65 | | 0,07 | | 2 | | Yes | | 14 809 779 | | | | 6 195 749 | | 8 614 030 |  |  |  |  |  |  |  |  |  |  |  |  |  |
| Astrologists | 0,32 | | 0,37 | | 0,05 | | 2 | | Yes | | 33 516 | | | | 22 956 | | 10 560 |  |  |  |  |  |  |  |  |  |  |  |  |  |
| Astronauts | - | | 0,23 | |  | |  | |  | | 0 | | | | 0 | | 0 |  |  |  |  |  |  |  |  |  |  |  |  |  |
| Astronomers | 0,25 | | 0,32 | | 0,07 | | 2 | | Yes | | 23 875 | | | | 17 907 | | 5 968 |  |  |  |  |  |  |  |  |  |  |  |  |  |
| Athletes | 0 | | 0,44 | | 0,44 | | 2 | |  | | 15 539 | | | | 15 539 | | 0 |  |  |  |  |  |  |  |  |  |  |  |  |  |
| Au pairs | 1 | | 0,77 | | -0,23 | | 2 | |  | | 121 896 | | | | 0 | | 121 896 |  |  |  |  |  |  |  |  |  |  |  |  |  |
| Auctioneers | 0,51 | | 0,31 | | -0,2 | | 2 | | Yes | | 49 884 | | | | 24 493 | | 25 391 |  |  |  |  |  |  |  |  |  |  |  |  |  |
| Authors | 0,21 | | 0,51 | | 0,3 | | 2 | |  | | 322 701 | | | | 255 043 | | 67 658 |  |  |  |  |  |  |  |  |  |  |  |  |  |
| Babies | 0,49 | | 0,52 | | 0,03 | | 5 | |  | | 795 494 | | | | 407 365 | | 388 129 |  |  |  |  |  |  |  |  |  |  |  |  |  |
| Babysitters | 1 | | 0,78 | | -0,22 | | 5 | |  | | 121 896 | | | | 0 | | 121 896 |  |  |  |  |  |  |  |  |  |  |  |  |  |
| Bakers | 0,45 | | 0,49 | | 0,04 | | 2 | |  | | 27 764 | | | | 15 196 | | 12 568 |  |  |  |  |  |  |  |  |  |  |  |  |  |
| Ballet dancers | 1 | | 0,71 | | -0,29 | | 2 | |  | | 11 061 | | | | 0 | | 11 061 |  |  |  |  |  |  |  |  |  |  |  |  |  |
| Bank clerks | 0,72 | | 0,43 | | -0,29 | | 2 | |  | | 134 631 | | | | 37 333 | | 97 298 |  |  |  |  |  |  |  |  |  |  |  |  |  |
| Bankers | 0,39 | | 0,32 | | -0,07 | | 2 | |  | | 246 598 | | | | 149 200 | | 97 398 |  |  |  |  |  |  |  |  |  |  |  |  |  |
| Barbers | 0,85 | | 0,2 | | -0,65 | | 2 | |  | | 174 669 | | | | 25 783 | | 148 886 |  |  |  |  |  |  |  |  |  |  |  |  |  |
| Bartenders | 0,55 | | 0,47 | | -0,08 | | 2 | |  | | 182 626 | | | | 82 640 | | 99 986 |  |  |  |  |  |  |  |  |  |  |  |  |  |
| Baseball players | - | | 0,2 | |  | |  | |  | | 0 | | | | 0 | | 0 |  |  |  |  |  |  |  |  |  |  |  |  |  |
| Basketball players | - | | 0,23 | |  | |  | |  | | 0 | | | | 0 | | 0 |  |  |  |  |  |  |  |  |  |  |  |  |  |
| Bass players | - | | 0,35 | |  | |  | |  | | 0 | | | | 0 | | 0 |  |  |  |  |  |  |  |  |  |  |  |  |  |
| Beauticians | 0,84 | | 0,84 | | 0 | | 2 | |  | | 70 153 | | | | 10 878 | | 59 275 |  |  |  |  |  |  |  |  |  |  |  |  |  |
| Bikers | - | | 0,3 | |  | |  | |  | | 0 | | | | 0 | | 0 |  |  |  |  |  |  |  |  |  |  |  |  |  |
| Bingo players | - | | 0,7 | |  | |  | |  | | 0 | | | | 0 | | 0 |  |  |  |  |  |  |  |  |  |  |  |  |  |
| Biologists | 0,57 | | 0,46 | | -0,11 | | 2 | |  | | 73 018 | | | | 31 641 | | 41 377 |  |  |  |  |  |  |  |  |  |  |  |  |  |
| Birth attendants | 1 | | 0,75 | | -0,25 | | 2 | |  | | 45 650 | | | | 0 | | 45 650 |  |  |  |  |  |  |  |  |  |  |  |  |  |
| Blacksmiths | 0,5 | | 0,21 | | -0,29 | | 2 | |  | | * | | | | * | | * |  |  |  |  |  |  |  |  |  |  |  |  |  |
| Bookkeepers | 0,71 | | 0,48 | | -0,23 | | 2 | |  | | 426 033 | | | | 123 467 | | 302 566 |  |  |  |  |  |  |  |  |  |  |  |  |  |
| Bosses | 0,45 | | 0,37 | | -0,08 | | 2 | | Yes | | 10 911 707 | | | | 5 978 344 | | 4 933 363 |  |  |  |  |  |  |  |  |  |  |  |  |  |
| Boutique owners | - | | 0,77 | |  | |  | |  | | 0 | | | | 0 | | 0 |  |  |  |  |  |  |  |  |  |  |  |  |  |
| Boxers | - | | 0,21 | |  | |  | |  | | 0 | | | | 0 | | 0 |  |  |  |  |  |  |  |  |  |  |  |  |  |
| Bricklayers | 0 | | 0,16 | | 0,16 | | 2 | |  | | 62 576 | | | | 62 576 | | 0 |  |  |  |  |  |  |  |  |  |  |  |  |  |
| Building contractors | 0 | | 0,21 | | 0,21 | | 2 | |  | | 230 380 | | | | 230 380 | | 0 |  |  |  |  |  |  |  |  |  |  |  |  |  |
| Bus drivers | 0,07 | | 0,25 | | 0,18 | | 2 | |  | | 97 472 | | | | 90 512 | | 6 960 |  |  |  |  |  |  |  |  |  |  |  |  |  |
| Butchers | 0 | | 0,21 | | 0,21 | | 2 | |  | | 38 184 | | | | 38 184 | | 0 |  |  |  |  |  |  |  |  |  |  |  |  |  |
| Butlers | 0,75 | | 0,2 | | -0,55 | | 2 | | Yes | | 56 732 | | | | 14 215 | | 42 517 |  |  |  |  |  |  |  |  |  |  |  |  |  |
| Cabinet ministers | 0,18 | | 0,28 | | 0,1 | | 6 | |  | | 22 | | | | 18 | | 4 |  |  |  |  |  |  |  |  |  |  |  |  |  |
| Cake decorators | 0,45 | | 0,72 | | 0,27 | | 2 | | Yes | | 27 764 | | | | 15 196 | | 12 568 |  |  |  |  |  |  |  |  |  |  |  |  |  |
| Camera operators | 0,18 | | 0,36 | | 0,18 | | 2 | |  | | 75 501 | | | | 61 944 | | 13 557 |  |  |  |  |  |  |  |  |  |  |  |  |  |
| Campers | - | | 0,41 | |  | |  | |  | | 0 | | | | 0 | | 0 |  |  |  |  |  |  |  |  |  |  |  |  |  |
| Canteen assistants | 0,73 | | 0,72 | | -0,01 | | 2 | |  | | 548 453 | | | | 148 984 | | 399 469 |  |  |  |  |  |  |  |  |  |  |  |  |  |
| Car mechanics | 0 | | 0,21 | | 0,21 | | 2 | |  | | 197 023 | | | | 197 023 | | 0 |  |  |  |  |  |  |  |  |  |  |  |  |  |
| Card players | - | | 0,34 | |  | |  | |  | | 0 | | | | 0 | | 0 |  |  |  |  |  |  |  |  |  |  |  |  |  |
| Care workers | 0,83 | | 0,72 | | -0,11 | | 2 | |  | | 696 433 | | | | 116 060 | | 580 373 |  |  |  |  |  |  |  |  |  |  |  |  |  |
| Caregivers | - | | 0,71 | |  | |  | |  | | 0 | | | | 0 | | 0 |  |  |  |  |  |  |  |  |  |  |  |  |  |
| Caretakers | 0,16 | | 0,31 | | 0,15 | | 2 | |  | | 89 418 | | | | 75 124 | | 14 294 |  |  |  |  |  |  |  |  |  |  |  |  |  |
| Carpenters | 0 | | 0,25 | | 0,25 | | 2 | |  | | 221 335 | | | | 221 335 | | 0 |  |  |  |  |  |  |  |  |  |  |  |  |  |
| Cartoonists | 0,54 | | 0,36 | | -0,18 | | 2 | | Yes | | 51 741 | | | | 24 048 | | 27 693 |  |  |  |  |  |  |  |  |  |  |  |  |  |
| Cashiers | 0,72 | | 0,59 | | -0,13 | | 2 | |  | | 589 824 | | | | 164 211 | | 425 613 |  |  |  |  |  |  |  |  |  |  |  |  |  |
| Cat owners | - | | 0,68 | |  | |  | |  | | 0 | | | | 0 | | 0 |  |  |  |  |  |  |  |  |  |  |  |  |  |
| Caterers | 0,55 | | 0,58 | | 0,03 | | 2 | |  | | 74 811 | | | | 33 458 | | 41 353 |  |  |  |  |  |  |  |  |  |  |  |  |  |
| Celebrities | - | | 0,54 | |  | |  | |  | | 0 | | | | 0 | | 0 |  |  |  |  |  |  |  |  |  |  |  |  |  |
| Chair persons | 0,29 | | 0,37 | | 0,08 | | 2 | |  | | 103 125 | | | | 73 690 | | 29 435 |  |  |  |  |  |  |  |  |  |  |  |  |  |
| Chamber-maids | 0,79 | | 0,82 | | 0,03 | | 2 | |  | | 570 459 | | | | 119 653 | | 450 806 |  |  |  |  |  |  |  |  |  |  |  |  |  |
| Cheerleaders | - | | 0,83 | |  | |  | |  | | 0 | | | | 0 | | 0 |  |  |  |  |  |  |  |  |  |  |  |  |  |
| Chefs | 0,2 | | 0,37 | | 0,17 | | 2 | |  | | 203 601 | | | | 163 354 | | 40 247 |  |  |  |  |  |  |  |  |  |  |  |  |  |
| Chemists | 0,24 | | 0,46 | | 0,22 | | 2 | |  | | 25 361 | | | | 19 373 | | 5 988 |  |  |  |  |  |  |  |  |  |  |  |  |  |
| Child educators | 0,74 | | 0,67 | | -0,07 | | 2 | |  | | 189 090 | | | | 49 247 | | 139 843 |  |  |  |  |  |  |  |  |  |  |  |  |  |
| Child-minders | 1 | | 0,78 | | -0,22 | | 2 | |  | | 121 896 | | | | 0 | | 121 896 |  |  |  |  |  |  |  |  |  |  |  |  |  |
| Children | 0,49 | | 0,51 | | 0,02 | | 5 | |  | | qq | | | | 6 885 963 | | 6 557 764 |  |  |  |  |  |  |  |  |  |  |  |  |  |
| Chiropractors | 0,78 | | 0,46 | | -0,32 | | 2 | | Yes | | 38 421 | | | | 8 413 | | 30 008 |  |  |  |  |  |  |  |  |  |  |  |  |  |
| Chocolate lovers | - | | 0,7 | |  | |  | |  | | 0 | | | | 0 | | 0 |  |  |  |  |  |  |  |  |  |  |  |  |  |
| Choreographers | 1 | | 0,59 | | -0,41 | | 2 | |  | | 11 061 | | | | 0 | | 11 061 |  |  |  |  |  |  |  |  |  |  |  |  |  |
| Cinema goers | - | | 0,53 | |  | |  | |  | | 0 | | | | 0 | | 0 |  |  |  |  |  |  |  |  |  |  |  |  |  |
| Clairvoyants | 0,32 | | 0,64 | | 0,32 | | 2 | | Yes | | 33 516 | | | | 22 956 | | 10 560 |  |  |  |  |  |  |  |  |  |  |  |  |  |
| Cleaners | 0,73 | | 0,71 | | -0,02 | | 2 | |  | | 629 366 | | | | 172 338 | | 457 028 |  |  |  |  |  |  |  |  |  |  |  |  |  |
| Clerks | 0,64 | | 0,42 | | -0,22 | | 2 | | Yes | | 4 681 448 | | | | 1 673 562 | | 3 007 886 |  |  |  |  |  |  |  |  |  |  |  |  |  |
| Clients | - | | 0,51 | |  | |  | |  | | 0 | | | | 0 | | 0 |  |  |  |  |  |  |  |  |  |  |  |  |  |
| Climbers | - | | 0,34 | |  | |  | |  | | 0 | | | | 0 | | 0 |  |  |  |  |  |  |  |  |  |  |  |  |  |
| Clinical psychologists | 0,77 | | 0,58 | | -0,19 | | 7 | |  | | 10 488 | | | | 2 379 | | 8 109 |  |  |  |  |  |  |  |  |  |  |  |  |  |
| Clowns | 0,31 | | 0,22 | | -0,09 | | 2 | | Yes | | 38 460 | | | | 26 668 | | 11 792 |  |  |  |  |  |  |  |  |  |  |  |  |  |
| Colonels | - | | 0,23 | |  | |  | |  | | 0 | | | | 0 | | 0 |  |  |  |  |  |  |  |  |  |  |  |  |  |
| Comedians | 0,31 | | 0,3 | | -0,01 | | 2 | | Yes | | 38 460 | | | | 26 668 | | 11 792 |  |  |  |  |  |  |  |  |  |  |  |  |  |
| Company directors | 0,38 | | 0,33 | | -0,05 | | 2 | |  | | 791 221 | | | | 494 011 | | 297 210 |  |  |  |  |  |  |  |  |  |  |  |  |  |
| Composers | 0,22 | | 0,35 | | 0,13 | | 2 | | Yes | | 37 898 | | | | 29 459 | | 8 439 |  |  |  |  |  |  |  |  |  |  |  |  |  |
| Computer programmers | 0,1 | | 0,26 | | 0,16 | | 2 | |  | | 253 184 | | | | 227 215 | | 25 969 |  |  |  |  |  |  |  |  |  |  |  |  |  |
| Computer specialists | 0,15 | | 0,26 | | 0,11 | | 2 | |  | | 1 038 130 | | | | 886 797 | | 151 333 |  |  |  |  |  |  |  |  |  |  |  |  |  |
| Computer technicians | 0,25 | | 0,26 | | 0,01 | | 2 | |  | | 117 244 | | | | 87 679 | | 29 565 |  |  |  |  |  |  |  |  |  |  |  |  |  |
| Conquerors | - | | 0,23 | |  | |  | |  | | 0 | | | | 0 | | 0 |  |  |  |  |  |  |  |  |  |  |  |  |  |
| Conservationists | - | | 0,5 | |  | |  | |  | | 0 | | | | 0 | | 0 |  |  |  |  |  |  |  |  |  |  |  |  |  |
| Construction workers | 0,01 | | 0,19 | | 0,18 | | 2 | |  | | 1 028 822 | | | | 1 021 929 | | 6 893 |  |  |  |  |  |  |  |  |  |  |  |  |  |
| Cooks | 0,2 | | 0,5 | | 0,3 | | 2 | |  | | 203 601 | | | | 163 354 | | 40 247 |  |  |  |  |  |  |  |  |  |  |  |  |  |
| Coordinators | 0,64 | | 0,5 | | -0,14 | | 2 | | Yes | | 5 638 460 | | | | 2 018 464 | | 3 619 996 |  |  |  |  |  |  |  |  |  |  |  |  |  |
| Counseling psychologists | 0,78 | | 0,69 | | -0,09 | | 7 | |  | | 1 706 | | | | 379 | | 1 327 |  |  |  |  |  |  |  |  |  |  |  |  |  |
| Counsellors | 0,63 | | 0,65 | | 0,02 | | 2 | |  | | 32 040 | | | | 11 809 | | 20 231 |  |  |  |  |  |  |  |  |  |  |  |  |  |
| Couriers | 0,16 | | 0,34 | | 0,18 | | 2 | |  | | 178 437 | | | | 149 477 | | 28 960 |  |  |  |  |  |  |  |  |  |  |  |  |  |
| Court reporters | 0,6 | | 0,46 | | -0,14 | | 2 | | Yes | | 505 398 | | | | 201 444 | | 303 954 |  |  |  |  |  |  |  |  |  |  |  |  |  |
| Craft workers | - | | 0,52 | |  | |  | |  | | 0 | | | | 0 | | 0 |  |  |  |  |  |  |  |  |  |  |  |  |  |
| Crane operators | 0 | | 0,19 | | 0,19 | | 2 | |  | | 6 987 | | | | 6 987 | | 0 |  |  |  |  |  |  |  |  |  |  |  |  |  |
| Criminals | 0,09 | | 0,31 | | 0,22 | | 8 | |  | | 204 354 | | | | 185 323 | | 19 031 |  |  |  |  |  |  |  |  |  |  |  |  |  |
| Cult members | - | | 0,42 | |  | |  | |  | | 0 | | | | 0 | | 0 |  |  |  |  |  |  |  |  |  |  |  |  |  |
| Curators | 1 | | 0,44 | | -0,56 | | 2 | |  | | 8 749 | | | | 0 | | 8 749 |  |  |  |  |  |  |  |  |  |  |  |  |  |
| Customers | 0,51 | | 0,58 | | 0,07 | | 5 | |  | | ‡ | | | | ‡ | | ‡ |  |  |  |  |  |  |  |  |  |  |  |  |  |
| Customs inspectors | 0,36 | | 0,4 | | 0,04 | | 2 | |  | | 77 722 | | | | 50 103 | | 27 619 |  |  |  |  |  |  |  |  |  |  |  |  |  |
| Customs officers | 0,51 | | 0,38 | | -0,13 | | 2 | | Yes | | 346 750 | | | | 171 554 | | 175 196 |  |  |  |  |  |  |  |  |  |  |  |  |  |
| Cyclists | - | | 0,41 | |  | |  | |  | | 0 | | | | 0 | | 0 |  |  |  |  |  |  |  |  |  |  |  |  |  |
| Dance instructors | 1 | | 0,7 | | -0,3 | | 2 | |  | | 11 061 | | | | 0 | | 11 061 |  |  |  |  |  |  |  |  |  |  |  |  |  |
| Dancers | 1 | | 0,65 | | -0,35 | | 2 | |  | | 11 061 | | | | 0 | | 11 061 |  |  |  |  |  |  |  |  |  |  |  |  |  |
| Data processors | 0,71 | | 0,38 | | -0,33 | | 2 | | Yes | | 35 293 | | | | 10 221 | | 25 072 |  |  |  |  |  |  |  |  |  |  |  |  |  |
| Database administrators | 0,25 | | 0,47 | | 0,22 | | 2 | | Yes | | 117 244 | | | | 87 679 | | 29 565 |  |  |  |  |  |  |  |  |  |  |  |  |  |
| Deacons | 0,18 | | 0,37 | | 0,19 | | 2 | |  | | 42 957 | | | | 35 391 | | 7 566 |  |  |  |  |  |  |  |  |  |  |  |  |  |
| Dental hygienists | 0,58 | | 0,57 | | -0,01 | | 2 | | Yes | | 40 055 | | | | 16 856 | | 23 199 |  |  |  |  |  |  |  |  |  |  |  |  |  |
| Dental technicians | 0,58 | | 0,5 | | -0,08 | | 2 | | Yes | | 40 055 | | | | 16 856 | | 23 199 |  |  |  |  |  |  |  |  |  |  |  |  |  |
| Dentists | 0,5 | | 0,38 | | -0,12 | | 2 | |  | | 42 737 | | | | 21 232 | | 21 505 |  |  |  |  |  |  |  |  |  |  |  |  |  |
| Deputies | - | | 0,41 | |  | |  | |  | | 0 | | | | 0 | | 0 |  |  |  |  |  |  |  |  |  |  |  |  |  |
| Designers | 0,31 | | 0,53 | | 0,22 | | 2 | | Yes | | 2 181 324 | | | | 1 513 702 | | 667 622 |  |  |  |  |  |  |  |  |  |  |  |  |  |
| Detectives | 0,19 | | 0,33 | | 0,14 | | 2 | | Yes | | 338 256 | | | | 272 870 | | 65 386 |  |  |  |  |  |  |  |  |  |  |  |  |  |
| Dieticians | 0,78 | | 0,65 | | -0,13 | | 2 | | Yes | | 43 348 | | | | 9 623 | | 33 725 |  |  |  |  |  |  |  |  |  |  |  |  |  |
| Diplomats | 0,27 | | 0,36 | | 0,09 | | 2 | | Yes | | 70 719 | | | | 51 382 | | 19 337 |  |  |  |  |  |  |  |  |  |  |  |  |  |
| Doctors of Philosophy | - | | 0,37 | |  | |  | |  | | 0 | | | | 0 | | 0 |  |  |  |  |  |  |  |  |  |  |  |  |  |
| Dog owners | - | | 0,49 | |  | |  | |  | | 0 | | | | 0 | | 0 |  |  |  |  |  |  |  |  |  |  |  |  |  |
| Door keepers | 0,26 | | 0,29 | | 0,03 | | 2 | | Yes | | 27 121 | | | | 19 988 | | 7 133 |  |  |  |  |  |  |  |  |  |  |  |  |  |
| Dressmakers | 1 | | 0,75 | | -0,25 | | 2 | |  | | 6 841 | | | | 0 | | 6 841 |  |  |  |  |  |  |  |  |  |  |  |  |  |
| Drivers | 0,46 | | 0,38 | | -0,08 | | 9 | |  | | 46 044 178 | | | | 24 822 514 | | 21 221 663 |  |  |  |  |  |  |  |  |  |  |  |  |  |
| Driving instructors | 0,22 | | 0,35 | | 0,13 | | 2 | |  | | 32 513 | | | | 25 412 | | 7 101 |  |  |  |  |  |  |  |  |  |  |  |  |  |
| Drug addicts | 0,26 | | 0,43 | | 0,17 | | 10 | |  | | 1 753 338 | | | | 1 303 182 | | 450 156 |  |  |  |  |  |  |  |  |  |  |  |  |  |
| Drug dealers | - | | 0,24 | |  | |  | |  | | 0 | | | | 0 | | 0 |  |  |  |  |  |  |  |  |  |  |  |  |  |
| Drummers | - | | 0,26 | |  | |  | |  | | 0 | | | | 0 | | 0 |  |  |  |  |  |  |  |  |  |  |  |  |  |
| Economists | 0,3 | | 0,33 | | 0,03 | | 2 | |  | | 35 422 | | | | 24 799 | | 10 623 |  |  |  |  |  |  |  |  |  |  |  |  |  |
| Editors | 0,43 | | 0,47 | | 0,04 | | 2 | |  | | 69 746 | | | | 39 870 | | 29 876 |  |  |  |  |  |  |  |  |  |  |  |  |  |
| Educational psychologists | 0,77 | | 0,63 | | -0,14 | | 7 | |  | | 4 251 | | | | 996 | | 3 255 |  |  |  |  |  |  |  |  |  |  |  |  |  |
| Electricians | 0 | | 0,21 | | 0,21 | | 2 | |  | | 470 749 | | | | 470 749 | | 0 |  |  |  |  |  |  |  |  |  |  |  |  |  |
| Embezzlers | - | | 0,37 | |  | |  | |  | | 0 | | | | 0 | | 0 |  |  |  |  |  |  |  |  |  |  |  |  |  |
| Engine-drivers | 0 | | 0,23 | | 0,23 | | 2 | |  | | 66 666 | | | | 66 666 | | 0 |  |  |  |  |  |  |  |  |  |  |  |  |  |
| Engineers | 0,19 | | 0,24 | | 0,05 | | 2 | | Yes | | 4 880 904 | | | | 3 934 343 | | 946 561 |  |  |  |  |  |  |  |  |  |  |  |  |  |
| Entertainers | 0,31 | | 0,46 | | 0,15 | | 2 | |  | | 38 460 | | | | 26 668 | | 11 792 |  |  |  |  |  |  |  |  |  |  |  |  |  |
| Environmentalists | - | | 0,54 | |  | |  | |  | | 0 | | | | 0 | | 0 |  |  |  |  |  |  |  |  |  |  |  |  |  |
| Estate agents | 0,51 | | 0,45 | | -0,06 | | 2 | | Yes | | 49 884 | | | | 24 493 | | 25 391 |  |  |  |  |  |  |  |  |  |  |  |  |  |
| Executives | 0,45 | | 0,36 | | -0,09 | | 2 | | Yes | | 2 627 685 | | | | 1 447 825 | | 1 179 860 |  |  |  |  |  |  |  |  |  |  |  |  |  |
| Executors | - | | 0,26 | |  | |  | |  | | 0 | | | | 0 | | 0 |  |  |  |  |  |  |  |  |  |  |  |  |  |
| Exercise instructors | 0,37 | | 0,49 | | 0,12 | | 2 | |  | | 78 858 | | | | 49 741 | | 29 117 |  |  |  |  |  |  |  |  |  |  |  |  |  |
| Exotic dancers | - | | 0,8 | |  | |  | |  | | 0 | | | | 0 | | 0 |  |  |  |  |  |  |  |  |  |  |  |  |  |
| Explorers | - | | 0,33 | |  | |  | |  | | 0 | | | | 0 | | 0 |  |  |  |  |  |  |  |  |  |  |  |  |  |
| Factory managers | - | | 0,28 | |  | |  | |  | | 0 | | | | 0 | | 0 |  |  |  |  |  |  |  |  |  |  |  |  |  |
| Fans | - | | 0,56 | |  | |  | |  | | 0 | | | | 0 | | 0 |  |  |  |  |  |  |  |  |  |  |  |  |  |
| Farmers | 0,1 | | 0,27 | | 0,17 | | 2 | |  | | 125 223 | | | | 112 255 | | 12 968 |  |  |  |  |  |  |  |  |  |  |  |  |  |
| Fashion models | 0,31 | | 0,7 | | 0,39 | | 2 | | Yes | | 38 460 | | | | 26 668 | | 11 792 |  |  |  |  |  |  |  |  |  |  |  |  |  |
| Fencers | - | | 0,36 | |  | |  | |  | | 0 | | | | 0 | | 0 |  |  |  |  |  |  |  |  |  |  |  |  |  |
| Figure skaters | - | | 0,67 | |  | |  | |  | | 0 | | | | 0 | | 0 |  |  |  |  |  |  |  |  |  |  |  |  |  |
| Film directors | 0,39 | | 0,31 | | -0,08 | | 2 | | Yes | | 16 624 | | | | 10 209 | | 6 415 |  |  |  |  |  |  |  |  |  |  |  |  |  |
| Film stars | - | | 0,51 | |  | |  | |  | | 0 | | | | 0 | | 0 |  |  |  |  |  |  |  |  |  |  |  |  |  |
| Financial advisers | 0,32 | | 0,36 | | 0,04 | | 2 | |  | | 179 225 | | | | 121 865 | | 57 360 |  |  |  |  |  |  |  |  |  |  |  |  |  |
| Financial analysts | 0,32 | | 0,35 | | 0,03 | | 2 | |  | | 179 225 | | | | 121 865 | | 57 360 |  |  |  |  |  |  |  |  |  |  |  |  |  |
| Firefighters | 0 | | 0,21 | | 0,21 | | 2 | |  | | 36 082 | | | | 36 082 | | 0 |  |  |  |  |  |  |  |  |  |  |  |  |  |
| Flight attendants | 0,62 | | 0,71 | | 0,09 | | 2 | |  | | 36 300 | | | | 13 692 | | 22 608 |  |  |  |  |  |  |  |  |  |  |  |  |  |
| Florists | 1 | | 0,77 | | -0,23 | | 2 | |  | | 10 918 | | | | 0 | | 10 918 |  |  |  |  |  |  |  |  |  |  |  |  |  |
| Flute players | - | | 0,64 | |  | |  | |  | | 0 | | | | 0 | | 0 |  |  |  |  |  |  |  |  |  |  |  |  |  |
| Football coaches | - | | 0,19 | |  | |  | |  | | 0 | | | | 0 | | 0 |  |  |  |  |  |  |  |  |  |  |  |  |  |
| Football players | 0,08 | | 0,21 | | 0,13 | | 11 | |  | | 3 183 000 | | | | 2 931 000 | | 252 000 |  |  |  |  |  |  |  |  |  |  |  |  |  |
| Forensic psychologists | 0,74 | | 0,48 | | -0,26 | | 7 | |  | | 993 | | | | 263 | | 730 |  |  |  |  |  |  |  |  |  |  |  |  |  |
| Forest rangers | 0 | | 0,25 | | 0,25 | | 2 | |  | | 7 405 | | | | 7 405 | | 0 |  |  |  |  |  |  |  |  |  |  |  |  |  |
| Fortune tellers | 0,32 | | 0,75 | | 0,43 | | 2 | | Yes | | 33 516 | | | | 22 956 | | 10 560 |  |  |  |  |  |  |  |  |  |  |  |  |  |
| Funeral directors | 0,31 | | 0,32 | | 0,01 | | 2 | | Yes | | 20 827 | | | | 14 417 | | 6 410 |  |  |  |  |  |  |  |  |  |  |  |  |  |
| Game wardens | 0 | | 0,33 | | 0,33 | | 2 | |  | | 20 867 | | | | 20 867 | | 0 |  |  |  |  |  |  |  |  |  |  |  |  |  |
| Gardeners | - | | 0,43 | |  | |  | |  | | 0 | | | | 0 | | 0 |  |  |  |  |  |  |  |  |  |  |  |  |  |
| General managers | 0,22 | | 0,42 | | 0,2 | | 2 | |  | | 548 026 | | | | 427 880 | | 120 146 |  |  |  |  |  |  |  |  |  |  |  |  |  |
| Generals | - | | 0,25 | |  | |  | |  | | 0 | | | | 0 | | 0 |  |  |  |  |  |  |  |  |  |  |  |  |  |
| Geologists | 0,25 | | 0,42 | | 0,17 | | 2 | |  | | 23 875 | | | | 17 907 | | 5 968 |  |  |  |  |  |  |  |  |  |  |  |  |  |
| Goalkeepers | 0,08 | | 0,2 | | 0,12 | | 11 | |  | | 289 364 | | | | 266 455 | | 22 909 |  |  |  |  |  |  |  |  |  |  |  |  |  |
| Goldsmiths | 0,3 | | 0,33 | | 0,03 | | 2 | | Yes | | 36 654 | | | | 25 831 | | 10 823 |  |  |  |  |  |  |  |  |  |  |  |  |  |
| Golfers | - | | 0,27 | |  | |  | |  | | 0 | | | | 0 | | 0 |  |  |  |  |  |  |  |  |  |  |  |  |  |
| Government officials | - | | 0,34 | |  | |  | |  | | 0 | | | | 0 | | 0 |  |  |  |  |  |  |  |  |  |  |  |  |  |
| Governors | - | | 0,33 | |  | |  | |  | | 0 | | | | 0 | | 0 |  |  |  |  |  |  |  |  |  |  |  |  |  |
| Graphic designers | 0,38 | | 0,42 | | 0,04 | | 2 | |  | | 85 731 | | | | 52 980 | | 32 751 |  |  |  |  |  |  |  |  |  |  |  |  |  |
| Groundkeepers | 0 | | 0,27 | | 0,27 | | 2 | |  | | 24 180 | | | | 24 180 | | 0 |  |  |  |  |  |  |  |  |  |  |  |  |  |
| Groupies | - | | 0,73 | |  | |  | |  | | 0 | | | | 0 | | 0 |  |  |  |  |  |  |  |  |  |  |  |  |  |
| Guards | 0,14 | | 0,23 | | 0,09 | | 2 | |  | | 185 170 | | | | 159 753 | | 25 417 |  |  |  |  |  |  |  |  |  |  |  |  |  |
| Guitar players | - | | 0,37 | |  | |  | |  | | 0 | | | | 0 | | 0 |  |  |  |  |  |  |  |  |  |  |  |  |  |
| Gymnasts | - | | 0,64 | |  | |  | |  | | 0 | | | | 0 | | 0 |  |  |  |  |  |  |  |  |  |  |  |  |  |
| Gynaecologists | 0,45 | | 0,5 | | 0,05 | | 2 | | Yes | | 240 895 | | | | 132 977 | | 107 918 |  |  |  |  |  |  |  |  |  |  |  |  |  |
| Hairdressers | 0,85 | | 0,7 | | -0,15 | | 2 | |  | | 174 669 | | | | 25 783 | | 148 886 |  |  |  |  |  |  |  |  |  |  |  |  |  |
| Head teachers | 0,32 | | 0,49 | | 0,17 | |  | |  | | 1680 | | | | 731 | | 949 |  |  |  |  |  |  |  |  |  |  |  |  |  |
| Heads of department | - | | 0,41 | |  | | 12 | |  | | 0 | | | | 0 | | 0 |  |  |  |  |  |  |  |  |  |  |  |  |  |
| Health visitors | - | | 0,66 | |  | |  | |  | | 0 | | | | 0 | | 0 |  |  |  |  |  |  |  |  |  |  |  |  |  |
| Heavy equipment operators | 0 | | 0,15 | | 0,15 | | 2 | |  | | 56 309 | | | | 56 309 | | 0 |  |  |  |  |  |  |  |  |  |  |  |  |  |
| Historians | 0,4 | | 0,39 | | -0,01 | | 2 | | Yes | | 16 202 | | | | 9 661 | | 6 541 |  |  |  |  |  |  |  |  |  |  |  |  |  |
| History professors | - | | 0,35 | |  | |  | |  | | 0 | | | | 0 | | 0 |  |  |  |  |  |  |  |  |  |  |  |  |  |
| Hitchhikers | - | | 0,37 | |  | |  | |  | | 0 | | | | 0 | | 0 |  |  |  |  |  |  |  |  |  |  |  |  |  |
| Homeless persons | - | | 0,33 | |  | |  | |  | | 0 | | | | 0 | | 0 |  |  |  |  |  |  |  |  |  |  |  |  |  |
| Horse riders | - | | 0,64 | |  | |  | |  | | 0 | | | | 0 | | 0 |  |  |  |  |  |  |  |  |  |  |  |  |  |
| Horse trainers | - | | 0,61 | |  | |  | |  | | 0 | | | | 0 | | 0 |  |  |  |  |  |  |  |  |  |  |  |  |  |
| Hospital orderlies | 0,8 | | 0,52 | | -0,28 | | 2 | | Yes | | 308 243 | | | | 62 274 | | 245 969 |  |  |  |  |  |  |  |  |  |  |  |  |  |
| Hotel guests | - | | 0,49 | |  | |  | |  | | 0 | | | | 0 | | 0 |  |  |  |  |  |  |  |  |  |  |  |  |  |
| Hotel managers | 0,52 | | 0,42 | | -0,1 | | 2 | |  | | 46 826 | | | | 22 584 | | 24 242 |  |  |  |  |  |  |  |  |  |  |  |  |  |
| Housekeepers | 1 | | 0,74 | | -0,26 | | 2 | |  | | 36 425 | | | | 0 | | 36 425 |  |  |  |  |  |  |  |  |  |  |  |  |  |
| Humanitarians | - | | 0,53 | |  | |  | |  | | 0 | | | | 0 | | 0 |  |  |  |  |  |  |  |  |  |  |  |  |  |
| Humanities professors | - | | 0,46 | |  | |  | |  | | 0 | | | | 0 | | 0 |  |  |  |  |  |  |  |  |  |  |  |  |  |
| Hunters | 0,78 | | 0,2 | | -0,58 | | 2 | | Yes | | 43 063 | | | | 9 422 | | 33 641 |  |  |  |  |  |  |  |  |  |  |  |  |  |
| Ice skaters | - | | 0,62 | |  | |  | |  | | 0 | | | | 0 | | 0 |  |  |  |  |  |  |  |  |  |  |  |  |  |
| Infant teachers | 0,87 | | 0,76 | | -0,11 | | 13 | |  | | 225 000 | | | | 28 600 | | 196 400 |  |  |  |  |  |  |  |  |  |  |  |  |  |
| Informants | - | | 0,42 | |  | |  | |  | | 0 | | | | 0 | | 0 |  |  |  |  |  |  |  |  |  |  |  |  |  |
| Inmates | 0,05 | | 0,31 | | 0,26 | | 14 | |  | | * | | | | * | | * |  |  |  |  |  |  |  |  |  |  |  |  |  |
| Innkeepers | 0,41 | | 0,41 | | 0 | | 2 | | Yes | | 38 813 | | | | 22 802 | | 16 011 |  |  |  |  |  |  |  |  |  |  |  |  |  |
| Interior decorators | 0,56 | | 0,62 | | 0,06 | | 2 | | Yes | | 56 333 | | | | 24 691 | | 31 642 |  |  |  |  |  |  |  |  |  |  |  |  |  |
| Interpreters | 0,6 | | 0,55 | | -0,05 | | 2 | | Yes | | 69 517 | | | | 27 828 | | 41 689 |  |  |  |  |  |  |  |  |  |  |  |  |  |
| Inventors | 0,53 | | 0,31 | | -0,22 | | 2 | | Yes | | 36 628 | | | | 17 092 | | 19 536 |  |  |  |  |  |  |  |  |  |  |  |  |  |
| IT consultants | 0,1 | | 0,29 | | 0,19 | | 2 | |  | | 170 287 | | | | 152 508 | | 17 779 |  |  |  |  |  |  |  |  |  |  |  |  |  |
| Jailors | - | | 0,33 | |  | |  | |  | | 0 | | | | 0 | | 0 |  |  |  |  |  |  |  |  |  |  |  |  |  |
| Janitors | 0,16 | | 0,27 | | 0,11 | | 2 | |  | | 89 418 | | | | 75 124 | | 14 294 |  |  |  |  |  |  |  |  |  |  |  |  |  |
| Jewelers | 0,37 | | 0,49 | | 0,12 | | 2 | | Yes | | 193 477 | | | | 122 698 | | 70 779 |  |  |  |  |  |  |  |  |  |  |  |  |  |
| Job Seekers | 0,34 | | 0,43 | | 0,09 | | 15 | |  | | * | | | | * | | * |  |  |  |  |  |  |  |  |  |  |  |  |  |
| Joggers | - | | 0,53 | |  | |  | |  | | 0 | | | | 0 | | 0 |  |  |  |  |  |  |  |  |  |  |  |  |  |
| Journalists | 0,43 | | 0,53 | | 0,1 | | 2 | |  | | 69 746 | | | | 39 870 | | 29 876 |  |  |  |  |  |  |  |  |  |  |  |  |  |
| Judges | 0,4 | | 0,34 | | -0,06 | | 2 | |  | | 24 999 | | | | 14 901 | | 10 098 |  |  |  |  |  |  |  |  |  |  |  |  |  |
| Jugglers | - | | 0,36 | |  | |  | |  | | 0 | | | | 0 | | 0 |  |  |  |  |  |  |  |  |  |  |  |  |  |
| Jurors | 0,51 | | 0,47 | | -0,04 | | 5 | |  | | ‡ | | | | ‡ | | ‡ |  |  |  |  |  |  |  |  |  |  |  |  |  |
| Karaoke singers | - | | 0,6 | |  | |  | |  | | 0 | | | | 0 | | 0 |  |  |  |  |  |  |  |  |  |  |  |  |  |
| Kids | 0,49 | | 0,5 | | 0,01 | | 5 | |  | | 9 585 959 | | | | 4 906 193 | | 4 679 766 |  |  |  |  |  |  |  |  |  |  |  |  |  |
| Killers | 0,06 | | 0,27 | | 0,21 | | 16 | |  | | 3 399 | | | | 3 198 | | 201 |  |  |  |  |  |  |  |  |  |  |  |  |  |
| Kindergarten teachers | 0,87 | | 0,79 | | -0,08 | | 13 | |  | | 225 000 | | | | 28 600 | | 196 400 |  |  |  |  |  |  |  |  |  |  |  |  |  |
| Knitters | - | | 0,81 | |  | |  | |  | | 0 | | | | 0 | | 0 |  |  |  |  |  |  |  |  |  |  |  |  |  |
| Lab technicians | - | | 0,44 | |  | |  | |  | | 0 | | | | 0 | | 0 |  |  |  |  |  |  |  |  |  |  |  |  |  |
| Landscape gardeners | 0,13 | | 0,4 | | 0,27 | | 2 | |  | | 145 579 | | | | 126 034 | | 19 545 |  |  |  |  |  |  |  |  |  |  |  |  |  |
| Law clerks | 0,63 | | 0,41 | | -0,22 | | 2 | | Yes | | 175 852 | | | | 65 186 | | 110 666 |  |  |  |  |  |  |  |  |  |  |  |  |  |
| Law professors | - | | 0,38 | |  | |  | |  | | 0 | | | | 0 | | 0 |  |  |  |  |  |  |  |  |  |  |  |  |  |
| Lawyers | 0,43 | | 0,41 | | -0,02 | | 2 | |  | | 64 660 | | | | 37 019 | | 27 641 |  |  |  |  |  |  |  |  |  |  |  |  |  |
| Leaders | - | | 0,36 | |  | |  | |  | | 0 | | | | 0 | | 0 |  |  |  |  |  |  |  |  |  |  |  |  |  |
| Librarians | 0,75 | | 0,67 | | -0,08 | | 2 | |  | | 30 844 | | | | 7 665 | | 23 179 |  |  |  |  |  |  |  |  |  |  |  |  |  |
| Lieutenants | - | | 0,25 | |  | |  | |  | | 0 | | | | 0 | | 0 |  |  |  |  |  |  |  |  |  |  |  |  |  |
| Lifeguards | 0,31 | | 0,38 | | 0,07 | | 2 | | Yes | | 59 895 | | | | 41 441 | | 18 454 |  |  |  |  |  |  |  |  |  |  |  |  |  |
| Lighthouse keepers | 0 | | 0,27 | | 0,27 | | 2 | |  | | 16 170 | | | | 16 170 | | 0 |  |  |  |  |  |  |  |  |  |  |  |  |  |
| Lodgers | - | | 0,42 | |  | |  | |  | | 0 | | | | 0 | | 0 |  |  |  |  |  |  |  |  |  |  |  |  |  |
| Lorry drivers | 0 | | 0,19 | | 0,19 | | 2 | |  | | 256 555 | | | | 256 555 | | 0 |  |  |  |  |  |  |  |  |  |  |  |  |  |
| Magician’s assistants | - | | 0,78 | |  | | 17 | | Yes | | 100 | | | | 95 | | 5 |  |  |  |  |  |  |  |  |  |  |  |  |  |
| Magicians | 0,05 | | 0,27 | | 0,22 | |  | |  | | * | | | | * | | * |  |  |  |  |  |  |  |  |  |  |  |  |  |
| Make up artists | 0,84 | | 0,78 | | -0,06 | | 2 | |  | | 70 153 | | | | 10 878 | | 59 275 |  |  |  |  |  |  |  |  |  |  |  |  |  |
| Managers | 0,45 | | 0,41 | | -0,04 | | 2 | | Yes | | 10 911 707 | | | | 5 978 344 | | 4 933 363 |  |  |  |  |  |  |  |  |  |  |  |  |  |
| Manicurists | 0,84 | | 0,84 | | 0 | | 2 | |  | | 70 153 | | | | 10 878 | | 59 275 |  |  |  |  |  |  |  |  |  |  |  |  |  |
| Mathematicians | 0,3 | | 0,32 | | 0,02 | | 2 | | Yes | | 35 422 | | | | 24 799 | | 10 623 |  |  |  |  |  |  |  |  |  |  |  |  |  |
| Mayors | - | | 0,31 | |  | |  | |  | | 0 | | | | 0 | | 0 |  |  |  |  |  |  |  |  |  |  |  |  |  |
| Mechanics | 0,04 | | 0,2 | | 0,16 | | 2 | | Yes | | 980 981 | | | | 941 924 | | 39 057 |  |  |  |  |  |  |  |  |  |  |  |  |  |
| Medical doctors | 0,45 | | 0,4 | | -0,05 | | 2 | | Yes | | 240 895 | | | | 132 977 | | 107 918 |  |  |  |  |  |  |  |  |  |  |  |  |  |
| Members of parliament | 0,23 | | 0,28 | | 0,05 | | 18 | |  | | 1 416 | | | | 1 094 | | 322 |  |  |  |  |  |  |  |  |  |  |  |  |  |
| Members of the armed forces | 0,1 | | 0,24 | | 0,14 | | 19 & 20 | |  | | 168 080 | | | | 151 630 | | 16 450 |  |  |  |  |  |  |  |  |  |  |  |  |  |
| Metallurgists | 0,12 | | 0,4 | | 0,28 | | 2 | | Yes | | 79 602 | | | | 69 812 | | 9 790 |  |  |  |  |  |  |  |  |  |  |  |  |  |
| Meteorologists | 0,25 | | 0,37 | | 0,12 | | 2 | | Yes | | 23 875 | | | | 17 907 | | 5 968 |  |  |  |  |  |  |  |  |  |  |  |  |  |
| Millionaires | - | | 0,34 | |  | |  | |  | | 0 | | | | 0 | | 0 |  |  |  |  |  |  |  |  |  |  |  |  |  |
| Miners | 0 | | 0,16 | | 0,16 | | 2 | |  | | 15 372 | | | | 15 372 | | 0 |  |  |  |  |  |  |  |  |  |  |  |  |  |
| Models | 0,31 | | 0,64 | | 0,33 | | 2 | | Yes | | 38 460 | | | | 26 668 | | 11 792 |  |  |  |  |  |  |  |  |  |  |  |  |  |
| Mountain climbers | - | | 0,33 | |  | |  | |  | | 0 | | | | 0 | | 0 |  |  |  |  |  |  |  |  |  |  |  |  |  |
| Murderers | 0,06 | | 0,28 | | 0,22 | | 16 | |  | | 3 399 | | | | 3 198 | | 201 |  |  |  |  |  |  |  |  |  |  |  |  |  |
| Musicians | - | | 0,47 | |  | |  | |  | | 0 | | | | 0 | | 0 |  |  |  |  |  |  |  |  |  |  |  |  |  |
| Nannies | 1 | | 0,82 | | -0,18 | | 2 | |  | | 121 896 | | | | 0 | | 121 896 |  |  |  |  |  |  |  |  |  |  |  |  |  |
| Neighbours | 0,51 | | 0,52 | | 0,01 | | 5 | |  | | ‡ | | | | ‡ | | ‡ |  |  |  |  |  |  |  |  |  |  |  |  |  |
| Neurologists | 0,45 | | 0,42 | | -0,03 | | 2 | | Yes | | 240 895 | | | | 132 977 | | 107 918 |  |  |  |  |  |  |  |  |  |  |  |  |  |
| Neurosurgeons | 0,45 | | 0,34 | | -0,11 | | 2 | | Yes | | 240 895 | | | | 132 977 | | 107 918 |  |  |  |  |  |  |  |  |  |  |  |  |  |
| News readers | - | | 0,49 | |  | |  | |  | | 0 | | | | 0 | | 0 |  |  |  |  |  |  |  |  |  |  |  |  |  |
| Novelists | 0,6 | | 0,54 | | -0,06 | | 2 | |  | | 69 517 | | | | 27 828 | | 41 689 |  |  |  |  |  |  |  |  |  |  |  |  |  |
| Nursery teachers | 0,87 | | 0,8 | | -0,07 | | 21 | |  | | 225 000 | | | | 28 600 | | 196 400 |  |  |  |  |  |  |  |  |  |  |  |  |  |
| Nurses | 0,89 | | 0,74 | | -0,15 | | 13 | |  | | * | | | | * | | * |  |  |  |  |  |  |  |  |  |  |  |  |  |
| Nutritionists | 0,57 | | 0,66 | | 0,09 | | 2 | | Yes | | 73 018 | | | | 31 641 | | 41 377 |  |  |  |  |  |  |  |  |  |  |  |  |  |
| Occupational therapists | 1 | | 0,61 | | -0,39 | | 2 | |  | | 36 584 | | | | 0 | | 36 584 |  |  |  |  |  |  |  |  |  |  |  |  |  |
| Oceanographers | 0,25 | | 0,47 | | 0,22 | | 2 | | Yes | | 23 875 | | | | 17 907 | | 5 968 |  |  |  |  |  |  |  |  |  |  |  |  |  |
| Office workers | - | | 0,51 | |  | |  | |  | | 0 | | | | 0 | | 0 |  |  |  |  |  |  |  |  |  |  |  |  |  |
| Orchestra conductors | - | | 0,31 | |  | |  | |  | | 0 | | | | 0 | | 0 |  |  |  |  |  |  |  |  |  |  |  |  |  |
| Orthodontists | 0,5 | | 0,43 | | -0,07 | | 2 | | Yes | | 42 737 | | | | 21 232 | | 21 505 |  |  |  |  |  |  |  |  |  |  |  |  |  |
| Palm readers | 0,32 | | 0,77 | | 0,45 | | 2 | | Yes | | 33 516 | | | | 22 956 | | 10 560 |  |  |  |  |  |  |  |  |  |  |  |  |  |
| Parachutists | - | | 0,36 | |  | |  | |  | | 0 | | | | 0 | | 0 |  |  |  |  |  |  |  |  |  |  |  |  |  |
| Parents | 0,5 | | 0,52 | | 0,02 | | 5 | |  | | * | | | | * | | * |  |  |  |  |  |  |  |  |  |  |  |  |  |
| Pastry chefs | 0,2 | | 0,5 | | 0,3 | | 2 | | Yes | | 203 601 | | | | 163 354 | | 40 247 |  |  |  |  |  |  |  |  |  |  |  |  |  |
| Patients | 0,59 | | 0,52 | | -0,07 | | 22 | |  | | 22 556 468 | | | | 9 305 776 | | 13 250 692 |  |  |  |  |  |  |  |  |  |  |  |  |  |
| Pawnbrokers | 0,38 | | 0,31 | | -0,07 | | 2 | | Yes | | 156 823 | | | | 96 867 | | 59 956 |  |  |  |  |  |  |  |  |  |  |  |  |  |
| Pedestrians | 0,51 | | 0,53 | | 0,02 | | 5 | |  | | ‡ | | | | ‡ | | ‡ |  |  |  |  |  |  |  |  |  |  |  |  |  |
| Pediatricians | - | | 0,52 | |  | |  | |  | | 0 | | | | 0 | | 0 |  |  |  |  |  |  |  |  |  |  |  |  |  |
| Pensioners | 0,56 | | 0,52 | | -0,04 | | 5 | |  | | 10 377 127 | | | | 4 594 538 | | 5 782 589 |  |  |  |  |  |  |  |  |  |  |  |  |  |
| Personal trainers | - | | 0,39 | |  | |  | |  | | 0 | | | | 0 | | 0 |  |  |  |  |  |  |  |  |  |  |  |  |  |
| Persons | 0,51 | | 0,5 | | -0,01 | | 5 | |  | | 63 182 178 | | | | 31 028 143 | | 32 154 035 |  |  |  |  |  |  |  |  |  |  |  |  |  |
| Pharmacists | 0,47 | | 0,53 | | 0,06 | | 23 | | Yes | | * | | | | * | | * |  |  |  |  |  |  |  |  |  |  |  |  |  |
| Philanthropists | - | | 0,48 | |  | |  | |  | | 0 | | | | 0 | | 0 |  |  |  |  |  |  |  |  |  |  |  |  |  |
| Photographers | - | | 0,46 | |  | |  | |  | | 0 | | | | 0 | | 0 |  |  |  |  |  |  |  |  |  |  |  |  |  |
| Physicians | 0,45 | | 0,37 | | -0,08 | | 2 | |  | | 240 895 | | | | 132 977 | | 107 918 |  |  |  |  |  |  |  |  |  |  |  |  |  |
| Physicists | 0,25 | | 0,32 | | 0,07 | | 2 | |  | | 23 875 | | | | 17 907 | | 5 968 |  |  |  |  |  |  |  |  |  |  |  |  |  |
| Physics students | - | | 0,28 | |  | |  | |  | | 0 | | | | 0 | | 0 |  |  |  |  |  |  |  |  |  |  |  |  |  |
| Physiotherapists | 0,71 | | 0,51 | | -0,2 | | 2 | |  | | 51 849 | | | | 15 126 | | 36 723 |  |  |  |  |  |  |  |  |  |  |  |  |  |
| Piano players | - | | 0,51 | |  | |  | |  | | 0 | | | | 0 | | 0 |  |  |  |  |  |  |  |  |  |  |  |  |  |
| Pilots | 0 | | 0,24 | | 0,24 | | 2 | |  | | 35 016 | | | | 35 016 | | 0 |  |  |  |  |  |  |  |  |  |  |  |  |  |
| Playgroup leaders | - | | 0,77 | |  | |  | |  | | 0 | | | | 0 | | 0 |  |  |  |  |  |  |  |  |  |  |  |  |  |
| Plumbers | 0 | | 0,21 | | 0,21 | | 2 | |  | | 176 165 | | | | 176 165 | | 0 |  |  |  |  |  |  |  |  |  |  |  |  |  |
| Poets | - | | 0,45 | |  | |  | |  | | 0 | | | | 0 | | 0 |  |  |  |  |  |  |  |  |  |  |  |  |  |
| Police inspectors | 0,18 | | 0,34 | | 0,16 | | 24 | |  | | 6 333 | | | | 5 165 | | 1 167 |  |  |  |  |  |  |  |  |  |  |  |  |  |
| Police officers | 0,27 | | 0,36 | | 0,09 | | 24 | |  | | 130 146 | | | | 94 868 | | 35 278 |  |  |  |  |  |  |  |  |  |  |  |  |  |
| Police sergeants | 0,2 | | 0,3 | | 0,1 | | 24 | |  | | 20 572 | | | | 16 537 | | 4 035 |  |  |  |  |  |  |  |  |  |  |  |  |  |
| Political activists | - | | 0,46 | |  | |  | |  | | 0 | | | | 0 | | 0 |  |  |  |  |  |  |  |  |  |  |  |  |  |
| Politicians | - | | 0,29 | |  | |  | |  | | 0 | | | | 0 | | 0 |  |  |  |  |  |  |  |  |  |  |  |  |  |
| Porters | 0 | | 0,27 | | 0,27 | | 2 | |  | | 10 563 | | | | 10 563 | | 0 |  |  |  |  |  |  |  |  |  |  |  |  |  |
| Postal workers | 0,16 | | 0,34 | | 0,18 | | 2 | |  | | 178 437 | | | | 149 477 | | 28 960 |  |  |  |  |  |  |  |  |  |  |  |  |  |
| Postmen | 0,16 | | 0,25 | | 0,09 | | 2 | |  | | 178 437 | | | | 149 477 | | 28 960 |  |  |  |  |  |  |  |  |  |  |  |  |  |
| Presidents | 0,05 | | 0,16 | | 0,11 | | 25 | |  | | 129 | | | | 122 | | 7 |  |  |  |  |  |  |  |  |  |  |  |  |  |
| Priests | 0,18 | | 0,16 | | -0,02 | | 2 | |  | | 42 957 | | | | 35 391 | | 7 566 |  |  |  |  |  |  |  |  |  |  |  |  |  |
| Primary school teachers | 0,87 | | 0,75 | | -0,12 | | 13 | |  | | 225 000 | | | | 28 600 | | 196 400 |  |  |  |  |  |  |  |  |  |  |  |  |  |
| Prison guards | 0,19 | | 0,26 | | 0,07 | | 2 | |  | | 45 931 | | | | 36 998 | | 8 933 |  |  |  |  |  |  |  |  |  |  |  |  |  |
| Prisoners | 0,05 | | 0,3 | | 0,25 | | 14 | |  | | * | | | | * | | * |  |  |  |  |  |  |  |  |  |  |  |  |  |
| Private detectives | 0,14 | | 0,31 | | 0,17 | | 2 | |  | | 185 170 | | | | 159 753 | | 25 417 |  |  |  |  |  |  |  |  |  |  |  |  |  |
| Probation officers | 1 | | 0,37 | | -0,63 | | 2 | |  | | 7 824 | | | | 0 | | 7 824 |  |  |  |  |  |  |  |  |  |  |  |  |  |
| Professional athletes | 0 | | 0,42 | | 0,42 | | 2 | |  | | 15 539 | | | | 15 539 | | 0 |  |  |  |  |  |  |  |  |  |  |  |  |  |
| Professors | - | | 0,38 | |  | |  | |  | | 0 | | | | 0 | | 0 |  |  |  |  |  |  |  |  |  |  |  |  |  |
| Projectionists | 0,18 | | 0,44 | | 0,26 | | 2 | |  | | 75 501 | | | | 61 944 | | 13 557 |  |  |  |  |  |  |  |  |  |  |  |  |  |
| Proofreaders | - | | 0,53 | |  | |  | |  | | 0 | | | | 0 | | 0 |  |  |  |  |  |  |  |  |  |  |  |  |  |
| Prostitutes | - | | 0,81 | |  | |  | |  | | 0 | | | | 0 | | 0 |  |  |  |  |  |  |  |  |  |  |  |  |  |
| Protestors | - | | 0,49 | |  | |  | |  | | 0 | | | | 0 | | 0 |  |  |  |  |  |  |  |  |  |  |  |  |  |
| Psychiatrists | 0,43 | | 0,55 | | 0,12 | | 26 | |  | | 11 400 | | | | 6 500 | | 4 900 |  |  |  |  |  |  |  |  |  |  |  |  |  |
| Psychology students | 0,8 | | 0,76 | | -0,04 | | 27 | |  | | * | | | | * | | * |  |  |  |  |  |  |  |  |  |  |  |  |  |
| Publishers | 0,39 | | 0,47 | | 0,08 | | 2 | |  | | 99 423 | | | | 60 914 | | 38 509 |  |  |  |  |  |  |  |  |  |  |  |  |  |
| Racing car drivers | - | | 0,19 | |  | |  | |  | | 0 | | | | 0 | | 0 |  |  |  |  |  |  |  |  |  |  |  |  |  |
| Radio announcers | - | | 0,43 | |  | |  | |  | | 0 | | | | 0 | | 0 |  |  |  |  |  |  |  |  |  |  |  |  |  |
| Radio technicians | - | | 0,34 | |  | |  | |  | | 0 | | | | 0 | | 0 |  |  |  |  |  |  |  |  |  |  |  |  |  |
| Rape victims | 0,96 | | 0,82 | | -0,14 | | 8 | |  | | 241 | | | | 10 | | 230 |  |  |  |  |  |  |  |  |  |  |  |  |  |
| Rapists | 0,01 | | 0,15 | | 0,14 | | 8 | |  | | 8 717 | | | | 8 636 | | 81 |  |  |  |  |  |  |  |  |  |  |  |  |  |
| Receptionists | 0,91 | | 0,75 | | -0,16 | | 2 | |  | | 239 172 | | | | 20 492 | | 218 680 |  |  |  |  |  |  |  |  |  |  |  |  |  |
| Relatives | 0,51 | | 0,5 | | -0,01 | | 5 | |  | | ‡ | | | | ‡ | | ‡ |  |  |  |  |  |  |  |  |  |  |  |  |  |
| Reporters | 0,52 | | 0,51 | | -0,01 | | 2 | | Yes | | 105 039 | | | | 50 091 | | 54 948 |  |  |  |  |  |  |  |  |  |  |  |  |  |
| Rescue workers | 0,85 | | 0,36 | | -0,49 | | 2 | | Yes | | 115 458 | | | | 16 950 | | 98 508 |  |  |  |  |  |  |  |  |  |  |  |  |  |
| Research scientists | 0,54 | | 0,42 | | -0,12 | | 2 | | Yes | | 39 664 | | | | 18 351 | | 21 313 |  |  |  |  |  |  |  |  |  |  |  |  |  |
| Road workers | 0 | | 0,19 | | 0,19 | | 2 | |  | | 24 589 | | | | 24 589 | | 0 |  |  |  |  |  |  |  |  |  |  |  |  |  |
| Roadies | - | | 0,38 | |  | |  | |  | | 0 | | | | 0 | | 0 |  |  |  |  |  |  |  |  |  |  |  |  |  |
| Romantic novelists | - | | 0,71 | |  | |  | |  | | 0 | | | | 0 | | 0 |  |  |  |  |  |  |  |  |  |  |  |  |  |
| Roofers | 0 | | 0,19 | | 0,19 | | 2 | |  | | 42 760 | | | | 42 760 | | 0 |  |  |  |  |  |  |  |  |  |  |  |  |  |
| Rubbish collectors | 0 | | 0,18 | | 0,18 | | 2 | |  | | 39 545 | | | | 39 545 | | 0 |  |  |  |  |  |  |  |  |  |  |  |  |  |
| Runners | - | | 0,46 | |  | |  | |  | | 0 | | | | 0 | | 0 |  |  |  |  |  |  |  |  |  |  |  |  |  |
| Sailors | - | | 0,27 | |  | |  | |  | | 0 | | | | 0 | | 0 |  |  |  |  |  |  |  |  |  |  |  |  |  |
| Sales assistants | 0,67 | | 0,64 | | -0,03 | | 2 | |  | | 1 460 917 | | | | 477 365 | | 983 552 |  |  |  |  |  |  |  |  |  |  |  |  |  |
| Salespersons | 0,64 | | 0,49 | | -0,15 | | 2 | |  | | 1 793 651 | | | | 645 270 | | 1 148 381 |  |  |  |  |  |  |  |  |  |  |  |  |  |
| Schoolchildrens | 0,49 | | 0,5 | | 0,01 | | 5 | |  | | 9 594 327 | | | | 4 914 589 | | 4 679 738 |  |  |  |  |  |  |  |  |  |  |  |  |  |
| Screenwriters | 0,6 | | 0,4 | | -0,2 | | 2 | |  | | 69 517 | | | | 27 828 | | 41 689 |  |  |  |  |  |  |  |  |  |  |  |  |  |
| Scuba divers | - | | 0,41 | |  | |  | |  | | 0 | | | | 0 | | 0 |  |  |  |  |  |  |  |  |  |  |  |  |  |
| Sculptors | 0,54 | | 0,45 | | -0,09 | | 2 | | Yes | | 51 741 | | | | 24 048 | | 27 693 |  |  |  |  |  |  |  |  |  |  |  |  |  |
| Secretaries | 0,94 | | 0,75 | | -0,19 | | 2 | |  | | 744 396 | | | | 45 639 | | 698 757 |  |  |  |  |  |  |  |  |  |  |  |  |  |
| Serial killers | - | | 0,23 | |  | |  | |  | | 0 | | | | 0 | | 0 |  |  |  |  |  |  |  |  |  |  |  |  |  |
| Sheriffs | - | | 0,23 | |  | |  | |  | | 0 | | | | 0 | | 0 |  |  |  |  |  |  |  |  |  |  |  |  |  |
| Shoemakers | 0 | | 0,34 | | 0,34 | | 2 | |  | | 6 305 | | | | 6 305 | | 0 |  |  |  |  |  |  |  |  |  |  |  |  |  |
| Shoplifters | - | | 0,46 | |  | |  | |  | | 0 | | | | 0 | | 0 |  |  |  |  |  |  |  |  |  |  |  |  |  |
| Shoppers | 0,51 | | 0,7 | | 0,19 | | 5 | |  | | ‡ | | | | ‡ | | ‡­­­­ |  |  |  |  |  |  |  |  |  |  |  |  |  |
| Singers | - | | 0,57 | |  | |  | |  | | 0 | | | | 0 | | 0 |  |  |  |  |  |  |  |  |  |  |  |  |  |
| Single parents | 0,9 | | 0,76 | | -0,14 | | 28 | |  | | 1 895 833 | | | | 180 808 | | 1 715 025 |  |  |  |  |  |  |  |  |  |  |  |  |  |
| Skiers | - | | 0,44 | |  | |  | |  | | 0 | | | | 0 | | 0 |  |  |  |  |  |  |  |  |  |  |  |  |  |
| Smugglers | - | | 0,32 | |  | |  | |  | | 0 | | | | 0 | | 0 |  |  |  |  |  |  |  |  |  |  |  |  |  |
| Snooker players | - | | 0,2 | |  | |  | |  | | 0 | | | | 0 | | 0 |  |  |  |  |  |  |  |  |  |  |  |  |  |
| Social workers | 0,75 | | 0,7 | | -0,05 | | 2 | |  | | 104 415 | | | | 26 025 | | 78 390 |  |  |  |  |  |  |  |  |  |  |  |  |  |
| Socialites | - | | 0,63 | |  | |  | |  | | 0 | | | | 0 | | 0 |  |  |  |  |  |  |  |  |  |  |  |  |  |
| Solarium users | - | | 0,55 | |  | |  | |  | | 0 | | | | 0 | | 0 |  |  |  |  |  |  |  |  |  |  |  |  |  |
| Soldiers | 0,08 | | 0,22 | | 0,14 | | 18 | |  | | * | | | | * | | * |  |  |  |  |  |  |  |  |  |  |  |  |  |
| Sound engineers | 0,18 | | 0,29 | | 0,11 | | 2 | | Yes | | 75 501 | | | | 61 944 | | 13 557 |  |  |  |  |  |  |  |  |  |  |  |  |  |
| Spectators | - | | 0,44 | |  | |  | |  | | 0 | | | | 0 | | 0 |  |  |  |  |  |  |  |  |  |  |  |  |  |
| Speech therapists | 1 | | 0,65 | | -0,35 | | 2 | |  | | 18 819 | | | | 0 | | 18 819 |  |  |  |  |  |  |  |  |  |  |  |  |  |
| Spies | - | | 0,38 | |  | |  | |  | | 0 | | | | 0 | | 0 |  |  |  |  |  |  |  |  |  |  |  |  |  |
| Sports coachs | 0,37 | | 0,33 | | -0,04 | | 2 | |  | | 78 858 | | | | 49 741 | | 29 117 |  |  |  |  |  |  |  |  |  |  |  |  |  |
| Statisticians | 0,3 | | 0,32 | | 0,02 | | 2 | |  | | 35 422 | | | | 24 799 | | 10 623 |  |  |  |  |  |  |  |  |  |  |  |  |  |
| Stockbrokers | 0 | | 0,28 | | 0,28 | | 2 | |  | | 35 213 | | | | 35 213 | | 0 |  |  |  |  |  |  |  |  |  |  |  |  |  |
| Strippers | - | | 0,69 | |  | |  | |  | | 0 | | | | 0 | | 0 |  |  |  |  |  |  |  |  |  |  |  |  |  |
| Structural engineers | 0,09 | | 0,26 | | 0,17 | | 2 | | Yes | | 71 062 | | | | 64 652 | | 6 410 |  |  |  |  |  |  |  |  |  |  |  |  |  |
| Students | 0,55 | | 0,55 | | 0 | | 29 | |  | | 1 367 310 | | | | 614 280 | | 753 030 |  |  |  |  |  |  |  |  |  |  |  |  |  |
| Sunbathers | - | | 0,68 | |  | |  | |  | | 0 | | | | 0 | | 0 |  |  |  |  |  |  |  |  |  |  |  |  |  |
| Supervisors | 0,49 | | 0,45 | | -0,04 | | 2 | |  | | 480 087 | | | | 245 423 | | 234 664 |  |  |  |  |  |  |  |  |  |  |  |  |  |
| Surfers | - | | 0,36 | |  | |  | |  | | 0 | | | | 0 | | 0 |  |  |  |  |  |  |  |  |  |  |  |  |  |
| Surgeons | 0,47 | | 0,33 | | -0,14 | | 2 | | Yes | | 366 687 | | | | 193 259 | | 173 428 |  |  |  |  |  |  |  |  |  |  |  |  |  |
| Suspects | - | | 0,36 | |  | |  | |  | | 0 | | | | 0 | | 0 |  |  |  |  |  |  |  |  |  |  |  |  |  |
| Swimmers | - | | 0,49 | |  | |  | |  | | 0 | | | | 0 | | 0 |  |  |  |  |  |  |  |  |  |  |  |  |  |
| Switchboard operators | 0,74 | | 0,52 | | -0,22 | | 2 | | Yes | | 18 606 | | | | 4 868 | | 13 738 |  |  |  |  |  |  |  |  |  |  |  |  |  |
| Synchronized swimmers | 1 | | 0,73 | | -0,27 | | 30 | |  | | * | | | | * | | * |  |  |  |  |  |  |  |  |  |  |  |  |  |
| Systems analysts | 0,11 | | 0,33 | | 0,22 | | 2 | |  | | 107 918 | | | | 95 612 | | 12 306 |  |  |  |  |  |  |  |  |  |  |  |  |  |
| Tailors | 1 | | 0,32 | | -0,68 | | 2 | |  | | 6 841 | | | | 0 | | 6 841 |  |  |  |  |  |  |  |  |  |  |  |  |  |
| Tap dancers | - | | 0,64 | |  | |  | |  | | 0 | | | | 0 | | 0 |  |  |  |  |  |  |  |  |  |  |  |  |  |
| Tarot card readers | 0,32 | | 0,73 | | 0,41 | | 2 | | Yes | | 33 516 | | | | 22 956 | | 10 560 |  |  |  |  |  |  |  |  |  |  |  |  |  |
| Tattooists | 0,84 | | 0,36 | | -0,48 | | 2 | | Yes | | 70 153 | | | | 10 878 | | 59 275 |  |  |  |  |  |  |  |  |  |  |  |  |  |
| Tax advisors | 0,4 | | 0,34 | | -0,06 | | 2 | |  | | 34 419 | | | | 20 509 | | 13 910 |  |  |  |  |  |  |  |  |  |  |  |  |  |
| Taxi drivers | 0,06 | | 0,2 | | 0,14 | | 2 | |  | | 192 400 | | | | 181 686 | | 10 714 |  |  |  |  |  |  |  |  |  |  |  |  |  |
| Taxidermists | 0,31 | | 0,35 | | 0,04 | | 2 | | Yes | | 20 827 | | | | 14 417 | | 6 410 |  |  |  |  |  |  |  |  |  |  |  |  |  |
| Technicians | 0,3 | | 0,35 | | 0,05 | | 2 | | Yes | | 4 574 040 | | | | 3 200 428 | | 1 373 612 |  |  |  |  |  |  |  |  |  |  |  |  |  |
| Technologists | 0,49 | | 0,34 | | -0,15 | | 2 | | Yes | | 426 717 | | | | 217 303 | | 209 414 |  |  |  |  |  |  |  |  |  |  |  |  |  |
| Teenagers | 0,49 | | 0,52 | | 0,03 | | 5 | |  | | 4 666 854 | | | | 2 391 358 | | 2 275 496 |  |  |  |  |  |  |  |  |  |  |  |  |  |
| Telephone operators | 1 | | 0,56 | | -0,44 | | 2 | |  | | 13 738 | | | | 0 | | 13 738 |  |  |  |  |  |  |  |  |  |  |  |  |  |
| Tennis players | - | | 0,47 | |  | |  | |  | | 0 | | | | 0 | | 0 |  |  |  |  |  |  |  |  |  |  |  |  |  |
| Terrorists | - | | 0,19 | |  | |  | |  | | 0 | | | | 0 | | 0 |  |  |  |  |  |  |  |  |  |  |  |  |  |
| Theatre directors | 0,34 | | 0,43 | | 0,09 | | 2 | |  | | 66 617 | | | | 44 286 | | 22 331 |  |  |  |  |  |  |  |  |  |  |  |  |  |
| Thieves | - | | 0,35 | |  | |  | |  | | 0 | | | | 0 | | 0 |  |  |  |  |  |  |  |  |  |  |  |  |  |
| Ticket inspectors | 0,26 | | 0,29 | | 0,03 | | 2 | | Yes | | 69 042 | | | | 51 048 | | 17 994 |  |  |  |  |  |  |  |  |  |  |  |  |  |
| Toddlers | 0,49 | | 0,5 | | 0,01 | | 5 | |  | | 2 354 608 | | | | 1 204 374 | | 1 150 234 |  |  |  |  |  |  |  |  |  |  |  |  |  |
| Toolmakers | 0 | | 0,24 | | 0,24 | | 2 | |  | | 16 047 | | | | 16 047 | | 0 |  |  |  |  |  |  |  |  |  |  |  |  |  |
| Tour guides | 0,43 | | 0,49 | | 0,06 | | 2 | |  | | 21 264 | | | | 12 162 | | 9 102 |  |  |  |  |  |  |  |  |  |  |  |  |  |
| Trade unionists | - | | 0,4 | |  | |  | |  | | 0 | | | | 0 | | 0 |  |  |  |  |  |  |  |  |  |  |  |  |  |
| Traffic engineers | 0,04 | | 0,26 | | 0,22 | | 2 | | Yes | | 256 398 | | | | 246 608 | | 9 790 |  |  |  |  |  |  |  |  |  |  |  |  |  |
| Train conductors | 0,38 | | 0,25 | | -0,13 | | 2 | | Yes | | 19 356 | | | | 11 922 | | 7 434 |  |  |  |  |  |  |  |  |  |  |  |  |  |
| Translators | 0,6 | | 0,57 | | -0,03 | | 2 | |  | | 69 517 | | | | 27 828 | | 41 689 |  |  |  |  |  |  |  |  |  |  |  |  |  |
| Travel agents | 0,75 | | 0,58 | | -0,17 | | 2 | |  | | 31 443 | | | | 7 768 | | 23 675 |  |  |  |  |  |  |  |  |  |  |  |  |  |
| Trumpet players | - | | 0,37 | |  | |  | |  | | 0 | | | | 0 | | 0 |  |  |  |  |  |  |  |  |  |  |  |  |  |
| Tuba players | - | | 0,43 | |  | |  | |  | | 0 | | | | 0 | | 0 |  |  |  |  |  |  |  |  |  |  |  |  |  |
| Typists | 0,71 | | 0,64 | | -0,07 | | 2 | |  | | 35 293 | | | | 10 221 | | 25 072 |  |  |  |  |  |  |  |  |  |  |  |  |  |
| Tyre fitters | 0 | | 0,21 | | 0,21 | | 2 | |  | | 14 770 | | | | 14 770 | | 0 |  |  |  |  |  |  |  |  |  |  |  |  |  |
| Undertakers | 0,31 | | 0,25 | | -0,06 | | 2 | |  | | 20 827 | | | | 14 417 | | 6 410 |  |  |  |  |  |  |  |  |  |  |  |  |  |
| Vets | 0,54 | | 0,5 | | -0,04 | | 2 | |  | | 19 125 | | | | 8 761 | | 10 364 |  |  |  |  |  |  |  |  |  |  |  |  |  |
| Vice chancellors | 0,43 | | 0,32 | | -0,11 | | 2 | | Yes | | 149 871 | | | | 84 767 | | 65 104 |  |  |  |  |  |  |  |  |  |  |  |  |  |
| Victims | 0,38 | | 0,63 | | 0,25 | | 8 | |  | | * | | | | * | | * |  |  |  |  |  |  |  |  |  |  |  |  |  |
| Violinists | - | | 0,62 | |  | |  | |  | | 0 | | | | 0 | | 0 |  |  |  |  |  |  |  |  |  |  |  |  |  |
| Waiters | 0,7 | | 0,45 | | -0,25 | | 2 | |  | | 215 970 | | | | 65 474 | | 150 496 |  |  |  |  |  |  |  |  |  |  |  |  |  |
| Wardens | 0,15 | | 0,35 | | 0,2 | | 2 | |  | | 268 729 | | | | 227 246 | | 41 483 |  |  |  |  |  |  |  |  |  |  |  |  |  |
| Weavers | 0,5 | | 0,7 | | 0,2 | | 2 | |  | | * | | | | * | | * |  |  |  |  |  |  |  |  |  |  |  |  |  |
| Wedding planners | 0,76 | | 0,78 | | 0,02 | | 2 | | Yes | | 63 744 | | | | 15 033 | | 48 711 |  |  |  |  |  |  |  |  |  |  |  |  |  |
| Weight lifters | - | | 0,24 | |  | |  | |  | | 0 | | | | 0 | | 0 |  |  |  |  |  |  |  |  |  |  |  |  |  |
| Welders | 0 | | 0,23 | | 0,23 | | 2 | |  | | 60 328 | | | | 60 328 | | 0 |  |  |  |  |  |  |  |  |  |  |  |  |  |
| Wine connoisseurs | - | | 0,38 | |  | |  | |  | | 0 | | | | 0 | | 0 |  |  |  |  |  |  |  |  |  |  |  |  |  |
| Wood carvers | 0 | | 0,25 | | 0,25 | | 2 | |  | | 34 077 | | | | 34 077 | | 0 |  |  |  |  |  |  |  |  |  |  |  |  |  |
| Wood workers | 0 | | 0,24 | | 0,24 | | 2 | |  | | 34 077 | | | | 34 077 | | 0 |  |  |  |  |  |  |  |  |  |  |  |  |  |
| Workers | 0,47 | | 0,42 | | -0,05 | | 31 | |  | | 45 530 210 | | | | 23 922 698 | | 21 607 512 |  |  |  |  |  |  |  |  |  |  |  |  |  |
| Wrestlers | - | | 0,2 | |  | |  | |  | | 0 | | | | 0 | | 0 |  |  |  |  |  |  |  |  |  |  |  |  |  |
| Writers | 0,6 | | 0,5 | | -0,1 | | 2 | |  | | 69 517 | | | | 27 828 | | 41 689 |  |  |  |  |  |  |  |  |  |  |  |  |  |
| X-ray technicians | 0,75 | | 0,47 | | -0,28 | | 2 | |  | | 23 675 | | | | 5 994 | | 17 681 |  |  |  |  |  |  |  |  |  |  |  |  |  |
| Youngsters | 0,49 | | 0,48 | | -0,01 | | 5 | |  | | 13 443 727 | | | | 6 885 963 | | 6 557 764 |  |  |  |  |  |  |  |  |  |  |  |  |  |
| Zoologists | 0,57 | | 0,49 | | -0,08 | | 2 | | Yes | | 73 018 | | | | 31 641 | | 41 377 |  |  |  |  |  |  |  |  |  |  |  |  |  |
|  | Notes. ‘-‘ represents currently unavailable ratios.  In numbers columns, 0 = no data, or sample too small for reliable estimate ;  * ratios only, available ; ‡ assumed same as persons | | | | | | | | | | | | | | | |  | | | |  | |  | |  | | |  | | |
| List of Sources | |  | |  | |  | | | |  | | |  |  | |  |  |  |  |  |  |  |  |  |  |  |  |  |  |  |
| 1. Financial Reporting Council (2013) | | | | | | 17. Nardi (1988) | | | | | | |  |  | |  |  |  |  |  |  |  |  |  |  |  |  |  |  |  |
| 2. ONS (2013b) | | | | | | 18. Ministry of Defence (2013) | | | | | | | |  | |  |  |  |  |  |  |  |  |  |  |  |  |  |  |  |
| 3. DASA (Navy) (2013) | | | | | | 19. UK Parliament (2014a) | | | | | | | |  | |  |  |  |  |  |  |  |  |  |  |  |  |  |  |  |
| 4. Health and Social Care Information Centre (2013) | | | | | | 20. UK Parliament (2014b) | | | |  | | | |  | |  |  |  |  |  |  |  |  |  |  |  |  |  |  |  |
| 5. ONS (2013a) | | | | | | 21. Shields, Hall, & Mamun (2011) | | | | | | | | | |  |  |  |  |  |  |  |  |  |  |  |  |  |  |  |
| 6. Government Digital Service (2014) | | | | | | 22. Health & Care Professions Council (2013) | | | | | | | | | |  |  |  |  |  |  |  |  |  |  |  |  |  |  |  |
| 7. Health & Care Professions Council (2012) | | | | | | 23. Hassell & Shann (2003) | | | | | | | | | |  |  |  |  |  |  |  |  |  |  |  |  |  |  |  |
| 8. Ministry of Justice (2011) | | | | | | 24. Home Office (2013b) | | | | | | | | | |  |  |  |  |  |  |  |  |  |  |  |  |  |  |  |
| 9. Department for Transport (2010) | | | | | | 25. Wikipedia (2014) | | | | | | | | | |  |  |  |  |  |  |  |  |  |  |  |  |  |  |  |
| 10. Home Office (2013a) | | | | | | 26. Royal College of Psychiatrists (2012) | | | | | | | | | |  |  |  |  |  |  |  |  |  |  |  |  |  |  |  |
| 11. Womens Sport and Fitness Foundation (2012) | | | | | | 27. Sanders, Sander, & Mercer (2009) | | | | | | | | | |  |  |  |  |  |  |  |  |  |  |  |  |  |  |  |
| 12. General Teaching Council for Wales (2012) | | | | | | 28. ONS (2013b) | | | | | | | | | |  |  |  |  |  |  |  |  |  |  |  |  |  |  |  |
| 13. Department for Education (2010) | | | | | | 29. Ratcliffe (2013) | | | | | | | | | |  |  |  |  |  |  |  |  |  |  |  |  |  |  |  |
| 14. Berman & Dar (2013) | | | | | | 30. Fédération Internationale de Natation (2013) | | | | | | | | | |  |  |  |  |  |  |  |  |  |  |  |  |  |  |  |
| 15. Joseph Rowntree Foundation (2013) | | | | | | 31. ONS (2014) | | | | | | | | | |  |  |  |  |  |  |  |  |  |  |  |  |  |  |  |
| 16. ONS (2013c) | | | |  | |  | | | | | | |  |  | |  |  |  |  |  |  |  |  |  |  |  |  |  |  |  |
